# Beyond the Scholl reaction – one-step planarization and edge chlorination of nanographenes by mechanochemistry†

**DOI:** 10.1039/d1ra07679e

**Published:** 2021-11-25

**Authors:** Daniel M. Baier, Sven Grätz, Babak Farhadi Jahromi, Sarah Hellmann, Konrad Bergheim, Wilm Pickhardt, Rochus Schmid, Lars Borchardt

**Affiliations:** Mechanochemistry Group, Faculty of Chemistry and Biochemistry, Ruhr-Universität Bochum Universitätsstraße 150 44780 Bochum Germany lars.borchardt@ruhr-uni-bochum.de; Computational Materials Chemistry Group, Faculty for Chemistry and Biochemistry, Ruhr-Universität Bochum Universitätsstraße 150 44780 Bochum Germany; Professur für Anorganische Chemie I, TU Dresden Bergstraße 66 D-01069 Dresden Germany

## Abstract

The edge chlorination of the benchmark nanographenes triphenylene and hexa-*peri*-hexabenzocoronene is conducted mechanochemically. This approach overcomes solubility limitations and eliminates the need for elaborate chlorination conditions. Additionally, the planarization of oligophenylenes and their edge-chlorination can be combined in a one-pot approach requiring as little as 60 minutes.

## Abstract 2

The edge chlorination of the benchmark nanographenes triphenylene and hexa-*peri*-hexabenzocoronene is conducted mechanochemically. Planarization and edge chlorination are combined which allows the preparation of chlorinated nanographenes in one step.

## Graphical abstract

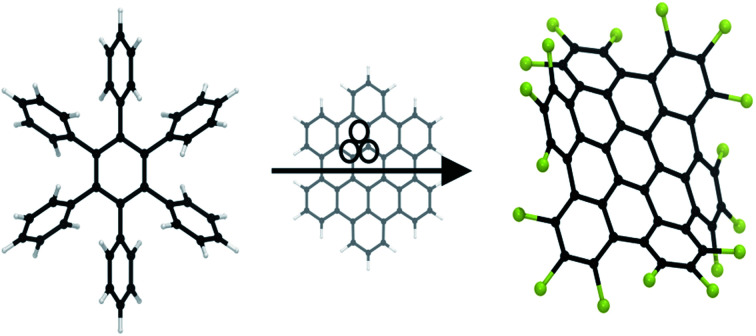

## Introduction

Since the discovery of graphene in 2004,^1^ graphene-based materials have been the focus of intensive research due to their versatile and outstanding properties resulting from the sp^2^-hybridized carbon surface.^2–4^ However, the absence of a bandgap in bulk graphene has so far prevented its use in many electronic applications. Nanographenes, which are spatially limited and virtually zero- (graphene quantum dots) or one-dimensional (graphene nanoribbons) possess band gaps, which depend on their size and edge configuration.^5^

**Fig. 1:**
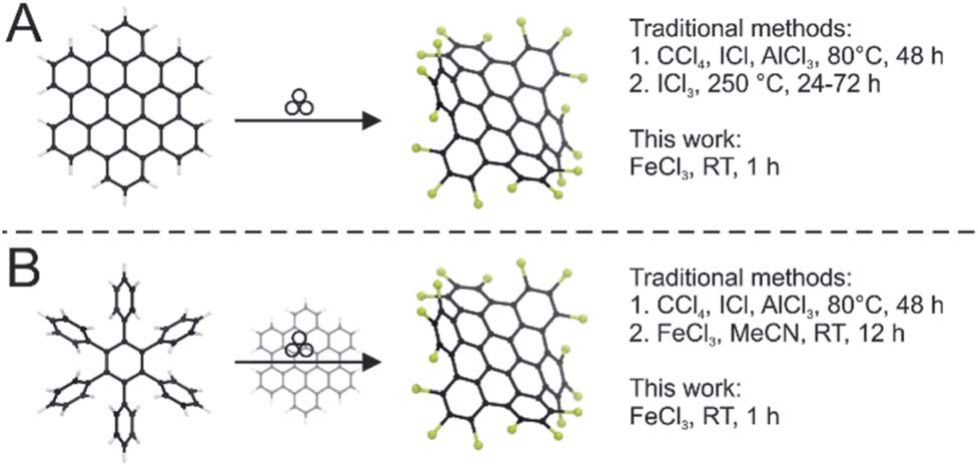
(A) Mechanochemical edge-chlorination reaction compared to established methods. (B) The one-step approach from hexaphenylbenzene to chlorinated HBC compared to the routes reported in the literature.

Such nanographenes can be prepared in two ways: “top-down” by nanolithography^6^ or unzipping of carbon nanotubes;^7^ or “bottom-up” by the targeted synthesis and planarization of precursor molecules.^8^ The former method has the drawback that the obtained size of the nanographene and its edge configuration can only be influenced insufficiently. To overcome this bottom-up pathways have been extensively studied. The most important reaction for this preparation of nanographenes is the Scholl reaction in which non-planar precursors are fully conjugated in the presence of a Lewis acid.^9^ It is also possible to perform planarization using halogenation reagents.^10^ The synthesis of such graphenes is mostly performed in solution. However, caused by strong pi–pi interactions nanographenes above a certain size become increasingly insoluble. Lately this lead to alternative reaction paths to nanographenes gaining more and more attention and importance.^11^ In the last decade mechanochemical reactions have regained the interest of the material and synthetic chemists. Ball milling has established itself as a powerful tool, offering at least as much flexibility as solvent-based processes.^12–16^ For many fields spanning everything from material synthesis,^17–22^ main group chemistry^23,24^ to organic chemistry,^25–28^ fullerene chemistry^29,30^ and lately polymer chemistry,^31^ solvent-free pathways have been developed in ball mills. We recently reported on the planarization of nanographenes using mechanochemistry.^11^ For these materials solubility is one of the major concerns and therefore the solvent-free pathway is offering an inherent advantage. Recently, the synthesis of curved nanographenes by means of mechanochemistry has also been reported.^32,33^ For subsequent applications, however, it is often necessary to convert the synthesized nanographenes into a soluble form. For this purpose, the graphenes can be chlorinated. The possibility of chlorination of electron-rich aromatic compounds by FeCl_3_ was first demonstrated by Niementowski in 1919.^34^ This chlorination often occurs as an undesirable side reaction.^35,36^ Chlorinated polyaromatic hydrocarbons are potentially toxic and should be avoided, but wherever they are needed the conventional synthetic methods for their genesis require exceedingly harsh and toxic conditions, with solvents such as CCl_4_, elevated temperatures and reaction times of several days (Fig. 1).^37^ Accordingly, there is a great need for a faster and more facile method. That aromatic substitution reactions towards aryl halides are indeed possible *via* mechanochemistry was first reported by Ondruschka and co-workers.^38^ They utilized oxone and the corresponding sodium halide to functionalize mesitylene with bromide and chloride groups. However, only small organic molecules and a maximum of three substitutions have been investigated.^38^ Interestingly, in our recent work on the planarization of nanographenes using Lewis acids we already noticed partial chlorination as a side reaction – especially under high energy conditions.^11^

In this study, we develop a protocol for the solvent-free edge-chlorination under mechanochemical conditions for benchmark nanographenes such as triphenylene (1) and hexabenzocoronene (3, HBC, Fig. 1). We investigate the influence of the milling parameters such as milling speed and time. The quality and structural homogeneity of the produced nanographenes are confirmed by MALDI-TOF mass spectrometry, IR-spectroscopy (Fig. S1 and S2†), X-ray diffraction (Fig. S3†) and UV-Vis absorption spectroscopy. In addition, an extensive computational study is conducted.

## Experimental section

In a typical synthesis, 0.1 g HBC 3 (0.192 mmol) and an excess of 1.9 g iron(iii) chloride (11.75 mmol, 61 eq.) were transferred into a 20 mL zirconium oxide grinding jar with 10 zirconium oxide 10 mm-diameter grinding balls (3.19 g each). The mixture was then milled for 60 min at 800 rpm in a Fritsch Pulverisette 7 premium line planetary ball mill. After the reaction, the grinding jar was opened and the reaction mixture was poured into water. The crude product was consequently washed with water, methanol and ethanol. The soluble fraction was extracted with CHCl_3_ which was consequently evaporated and the solid was dried at 80 °C. Chlorinated HBC 4 was obtained as a dark red solid.

For a typical one-pot reaction 0.1 g hexaphenylbenzene 5 (0.187 mmol) and 2.18 g iron(iii) chloride (13.44 mmol, 72 eq.) were transferred into a 20 mL zirconium oxide grinding jar with 10 zirconium oxide 10 mm-diameter grinding balls (3.19 g each). The mixture was then milled for 60 min at 800 rpm in a Fritsch Pulverisette 7 premium line planetary ball mill. After the reaction, the grinding jar was opened and the reaction mixture was poured into water. The crude product was consequently washed with water, methanol and ethanol. The soluble fraction was extracted with CHCl_3_ which was consequently evaporated and the solid was dried at 80 °C. Chlorinated HBC 4 was obtained as a dark red solid.

Matrix assisted laser desorption ionization time of flight mass spectroscopy (MALDI-TOF) was carried out on a Bruker Autoflex Speed spectrometer using a 337 nm nitrogen laser or on a Bruker Ultraflex 3 with 7,7,8,8-tetracyanoquinodimethane (TCNQ) or *trans*-2-[3-(4-*tert*-butylphenyl)-2-methyl-2-propenylidene]malononitrile (DCTB) as matrix if not indicated otherwise. UV/Vis spectra were measured on a UV-Vis-NIR Spectrophotometer Cary 5000 or on a Shimadzu UV-1900i at room temperature using a 10 mm quartz cell. Infrared (IR) spectra were measured on a Shimadzu IRSpirit Fourier transform infrared spectrometer with a single reflection ATR unit. Powder X-ray diffractograms (PXRDs) were measured on a Bruker D2 Phaser diffractometer with a LynxEye detector at an acceleration voltage of 30 kV and an emission current of 10 mA using Cu K-α radiation with a wavelength of *λ* = 1.54184 Å. The diffractograms were recorded from 10–70° (2*θ*).

All DFT calculations were performed with the TURBOMOLE V7.3 (ref. 39) program package, at the B3LYP^40,41^-D3(BJ)^42,43^/def2-TZVP^44^ level of theory on an “m5” grid^45^ and using the resolution-of-identity approximation for the computation of the Coulomb integrals (RI-*J*).^46,47^ All GFN2-xTB calculations were performed with the LAMMPS^48^ program package with a python-based interface to the xtb^49^ program package, that is implemented in the in-house developed pylmps wrapper, which was used to drive the calculations. Illustrations of molecular structures were generated using the VMD^50^ program package and the Tachyon ray tracing library.^51^

## Results and discussion

### Chlorination of triphenylene

**Fig. 2:**
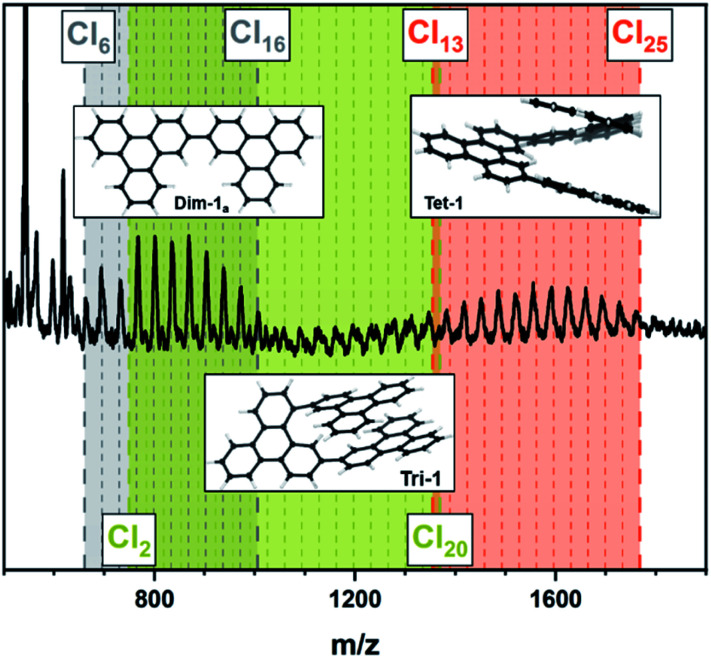
MALDI-TOF spectra of Table 1, entry 1; fraction soluble in CHCl_3_. The colours indicate the different oligomers: grey: dimer; green: trimer; orange: tetramer. The dashed lines indicate the degree of chlorination. The minimum and maximum number of substituted chlorine atoms are indicated separately for each oligomer and are indicated separately by the thick dashed lines. The optimized structures of the energetically most favourable constitutional isomers according to DFT are given as inset for each oligomer.

We identified 1 as a suitable model compound and conducted the reaction with an excess of FeCl_3_ at a milling frequency of 800 rpm for 60 minutes in a planetary ball mill (Table 1, entry 1). After washing the obtained product was analyzed by MALDI-TOF (Fig. 2).

**Table 1:** Reaction conditions and yields of the chlorinated nanographenes syntheses; reaction conditions if not stated otherwise: 800 rpm, 10 × 10 mm balls, ZrO_2_, 60 min

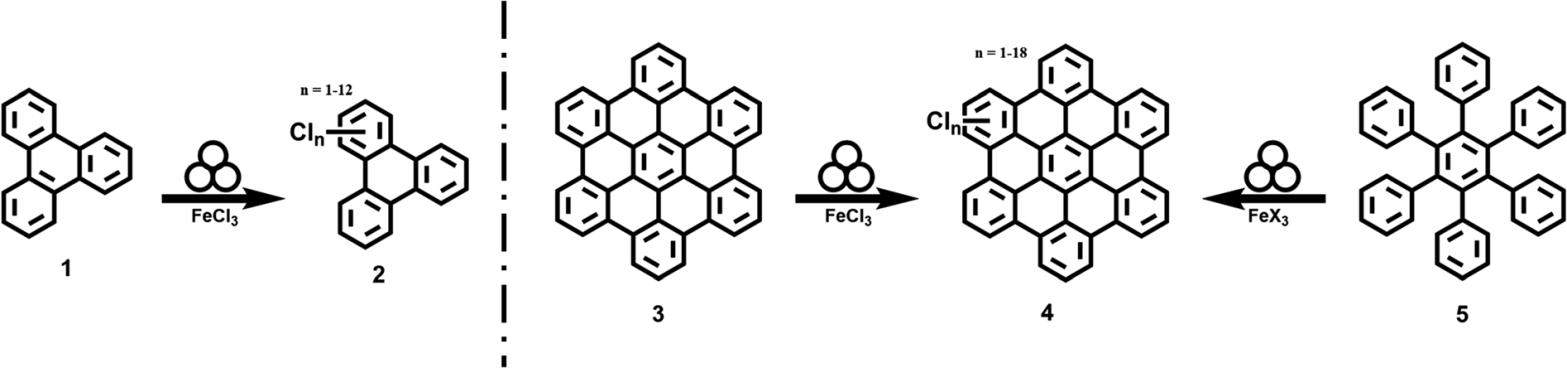						
Entry	Substrate	FeX_3_	Mill	Time (min)	Sol. fraction^a^ (%)	Yield^b^ (%)
1	1	FeCl_3_	P7	60	100	52
2	3	FeCl_3_	P7	60	81	49
3	3	FeCl_3_	P7	720	22	22
4^c^	3	FeCl_3_	P7	60	51	35
5^d^	3	FeCl_3_	EMAX	60	55	28
6	5	FeCl_3_	P7	60	75^e^	64^e^
7	5	FeCl_3_	P7	720	41	56
8	5	FeBr_3_	P7	60	—	18
9	5	FeBr_3_	P7	720	30	15

a After the reaction, the crude reaction mixture is taken up in water, filtered and washed with MeOH and EtOH. The residue obtained is dried overnight at 80 °C and then extracted with CHCl_3_. This is the soluble fraction.

b Yield calculated for the soluble fraction in regard to perhalogenated nanographenes.

c Only 2.5 wt% substrate.

d Reaction conditions: 1200 rpm, 50 mL ZrO_2_ vessel, 25 × 10 mm balls, 60 min.

e The reaction was performed four times separately and the average value is given.

At first glance the spectrum looks chaotic but an in-depth analysis reveals the formation of oligomers with varying degrees of chlorination. The dimer (grey area) is chlorinated up to 16 times with the highest intensity found at a chlorination degree of 12. The chlorination of the trimer (green area) is in the range between 2 and 20 chlorine atoms and the signal intensity is significantly lower compared to the dimer. Finally, a chlorination of 13 to 25 chlorine atoms can be found for the tetramer (orange area), whereby the signal intensity is above that of the trimer. This may be the result of a formation of the tetramer directly from the dimer similar to a step growth polymerization. Perchlorination could not be found for any of the observed oligomers. In order to investigate the structure of the oligomers and the degree of chlorination more closely, we carried out calculations employing the extended tight-binding GFN2-xTB method,^52^ as well as dispersion corrected Density Functional Theory (DFT). Only those oligomers were considered that link the triphenylene units with a single C–C bond. Among the three possible dimers, the dimer Dim-1_a_ (Fig. S15†) was computed to be the most stable constitutional isomer. This is in line with expectations, as the triphenylene units are furthest apart here. In contrast, for the favoured trimer Tri-1, the two monomers are attached close to each other at the central triphenylene, maximizing pi-stacking, which obviously compensates for the steric effects to a certain extent. However, it must be noted that these preferences are purely thermodynamic, whereas kinetic effects would imply the determination of reaction mechanisms and barriers, which would require much more computational effort. The trend of face-to-face stacking continues in the tetramer. The most stable tetramer, Tet-1, arranges itself in such a way that the two terminal monomer units have as large an overlap as possible. This structure represents a compound of two Dim-1_a_ units. This supports our hypothesis that the tetramer arises directly from the dimer in a kind of step growth polymerisation.

To investigate the degree of chlorination, we calculated the equilibrium structures of a reasonable selection out of all possible isomers for all degrees of chlorination of Dim-1_a_ (see ESI† for details on the selection of isomers). If we compare the energetically most favourable structure for each degree of chlorination, we notice that the system becomes more stable with each chlorine atom introduced (see Fig. S19†). However, it is noticeable that this stabilization is greatest for the first chlorine atoms introduced and decreases as chlorination progresses. In particular, a drop in the energy gain can be seen at a substitution level of 10, beyond which every further chlorination is accompanied by a more drastic distortion of the dimer structure, due to increasing steric repulsion. These results fit well with our experimental data in which the signals for degrees of chlorination 9–12 are most intense for the dimer. The absence of perchlorinated species can thus be explained on the one hand by the preferential oligomerisation and on the other hand by the decreasing stabilisation per chlorination for higher degrees of chlorination.

Since the Scholl reaction as a tool for polymer synthesis is well-known and has already been studied intensively by us,^22^ the appearance of oligomers of chlorinated triphenylene can be easily explained. Indeed, the susceptibility of small unsubstituted nanographenes such as 1 to oligomerisation under Scholl conditions is known from the literature.^53^

### Chlorination of HBC

**Fig. 3:**
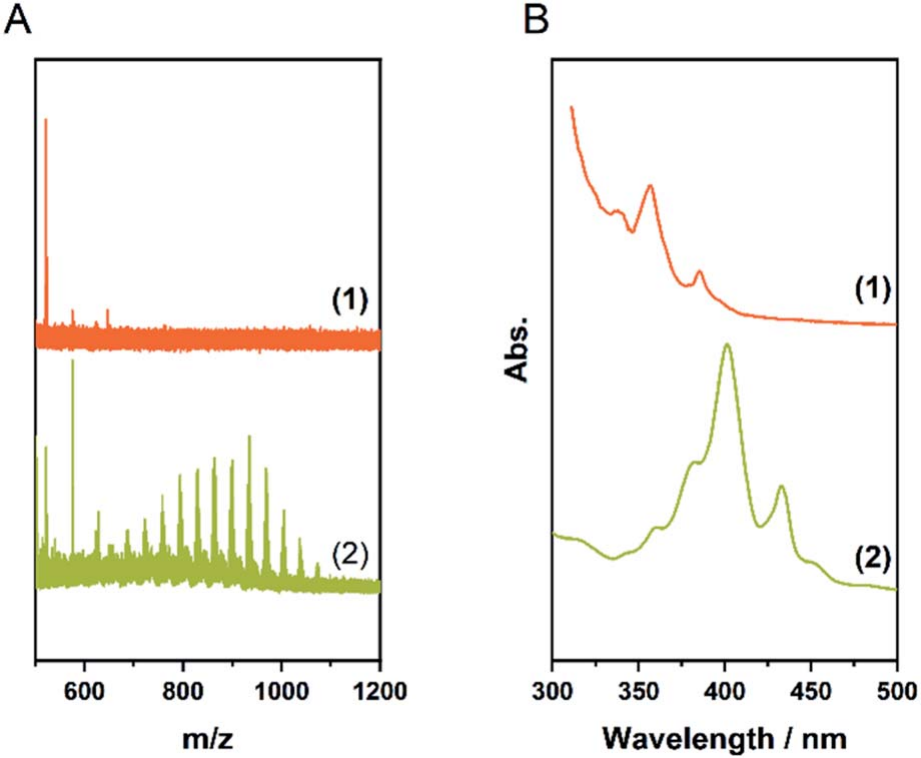
(A) MALDI-TOF spectra of Table 1, entry 2; (1) fraction insoluble in CHCl_3_ (2) soluble fraction. (B) UV/Vis spectra in toluene of the soluble fraction (2) compared to the pristine HBC (1).

Since larger nanographenes are known to be less likely to undergo oligomerization under the given conditions we turned to 3 as a well-established model compound.^11^ We applied the parameters described above. This led to a product with a high degree of chlorination in the CHCl_3_ soluble fraction. The chlorinated HBC shows the highest intensity in the MALDI-TOF spectrum at a number of 12 chlorine atoms introduced and a maximum substitution of up to 16, thus being already close to perchlorination (Fig. 3A and Table 1, entry 2). Furthermore, no oligomerisation was observed. This confirms our assumption that larger nanographenes are less susceptible to this side reaction under our conditions. The UV/Vis spectrum of the soluble fraction (Fig. 3B) shows a bathochromic shift compared to the pristine HBC and is in good agreement with the spectrum reported for perchlorinated 3.^37^ The comparison of the IR spectra (Fig. S2†) of the soluble fraction and HBC clearly show the formation of chlorinated HBC.^54^ The characteristic signals of HBC at about 740 and 760 cm^−1^ disappear in chlorinated HBC. Only a broadened band can be detected. These signals belong to the out-of-plane bending of the C–H bonds and their disappearance corresponds to the expectations in the course of chlorination. At the same time, a signal at 806 cm^−1^ appears in the chlorinated HBC which can be assigned to the out-of-phase vibration of the C–Cl bond and thus also speaks for the formation of C–Cl bonds. Furthermore, signals at 1175, 1258, 1291 and 1315 cm^−1^ can be seen in the chlorinated HBC, which are in good agreement with the literature, while the characteristic signals for HBC disappear at 1224, 1373 and 1490 cm^−1^.

### Influence of the milling parameters

In further experiments neither the increase of the milling time nor the doubling of the amount of FeCl_3_, (Table 1, entries 3 and 4) could push the system further towards perchlorination.

The increase in milling time even reduced the yield of soluble product likely due the formation of insoluble oligo- and polymers by a Scholl-polymerization. To avoid this, we maintained a short reaction time but aimed to increase the energy input. For this reason, we transferred our reaction to an EMAX high-energy ball mill. That allows an increase of the grinding frequency. This approach yielded perchlorinated HBC in small amounts while the average degree of chlorination was 7. The yield of soluble product was again rather low (Table 1, entry 5). Furthermore, this higher energy input led to dimerization of the chlorinated species (Fig. S4†) indicating that dimerization is also a side reaction for the chlorination of HBC once the kinetic energy is sufficiently high.

### Influence of the Lewis acid

Besides the process parameters of the ball mill the underlying chemistry plays a major role in the edge-chlorination reaction. For this reason, we investigated other Lewis acids as well as alternative chlorination methods for the mechanochemical edge-chlorination. While the former did not produce comparable results to FeCl_3_, we were able to successfully chlorinate 3 with oxone and obtained a soluble fraction of 83% and a yield of 43% (Table S1, entry S8†). This is the first time a larger nanographene has been chlorinated using this method.^38^ Although the yield is about 20% lower than with FeCl_3_, this method can be a more sustainable approach for the chlorination of nanographenes as it is completely transition metal free. However, only up to 15 chlorine atoms could be introduced, with the highest intensity being 10 substitutions. Thus, the oxone approach lags behind the FeCl_3_ approach in terms of both yield and degree of chlorination (for further details, see ESI Sections 3–5†).

### One-pot chlorination of HBC

**Fig. 4:**
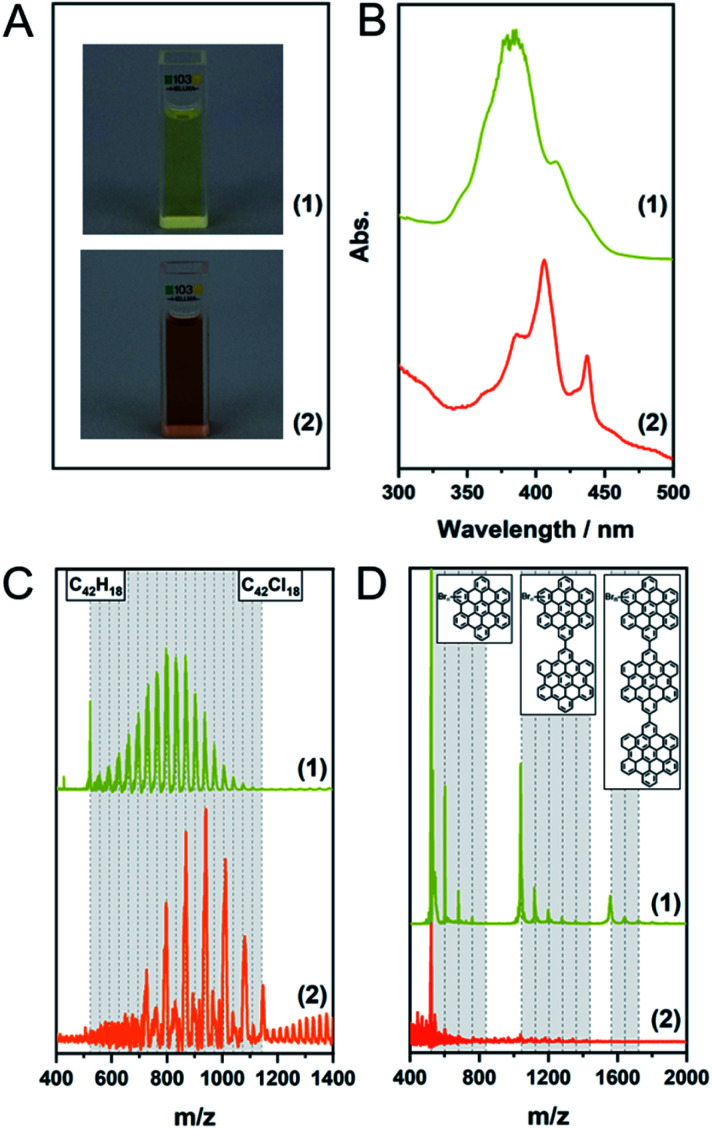
(A) Photos of the toluene solutions, (B) UV/Vis spectra in toluene, (C) MALDI-TOF spectra of Table 1, entries 6 (1) and 7 (2). The dashed lines indicate the degree of chlorination. (D) MALDI-TOF spectra of Table 1, entry 8, (1): insoluble fraction, (2): fraction soluble in CHCl_3_. The grey areas indicate the different oligomers. The dashed lines indicate the degree of bromination.

Finally, we combined planarization and edge-chlorination in a one-pot synthesis directly starting from the oligophenylene precursor 5. This reduces the number of necessary processing steps and directly yields a soluble molecule. According to the results presented so far, we investigated the use of FeCl_3_ to achieve a one-pot synthesis to 4 (Table 1, entries 6 and 7). The MALDI-TOF spectra as well as photographs of the toluene solutions and their UV/Vis spectra of the experiments after one and 12 hours are shown in Fig. 4A–C. Already after a reaction time of one hour, 4 could be obtained and up to 16 chlorine atoms could be introduced while the maximum is 9. This value could be shifted to 12 by increasing the reaction time to 12 h. This extended milling time also forced the system to perchlorination. Furthermore, the UV/Vis spectra show that after one hour a partially chlorinated HBC is already present which is bathochromically shifted compared to the pristine HBC (Fig. 3), while the spectrum after 12 hours is in perfect agreement with the literature spectrum of the perchlorinated HBC.^37^

**Fig. 5:**
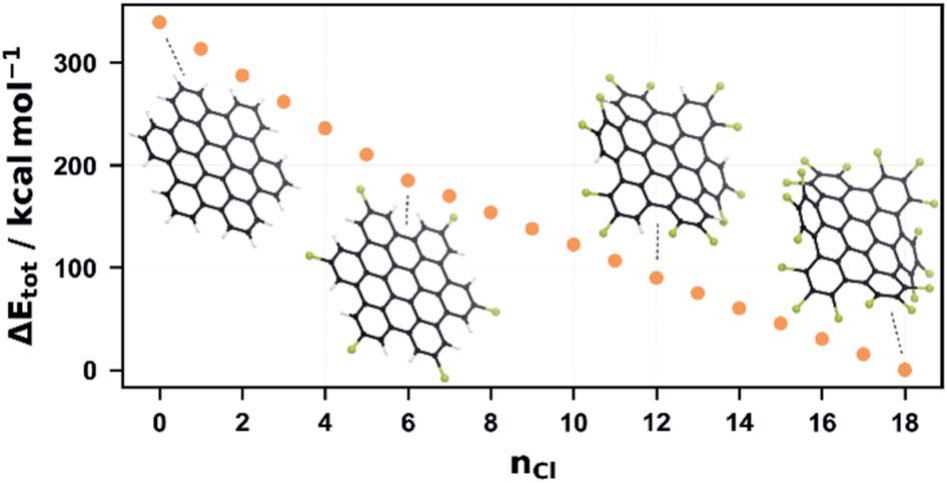
Relative molecular energies of the most favorable chlorinated isomer derived from HBC per degree of chlorination, obtained from DFT calculations, as well as the DFT-optimized geometries of the isomers with *n*_Cl_ = 0, 6, 18 and 18.

As for 1, we have also undertaken a computational study for this system. The molecular structures of all possible chlorinated HBC species were optimized on the GFN2-xTB level and the most energetically favourable structure was determined for each degree of chlorination, which was then further refined using DFT (Fig. 5). Similar to the dimer, the system becomes more energetically favourable with each chlorination step and, as with the dimer, this energy gain is highest for the first chlorinations and decreases with each chlorine atom introduced. In contrast to the dimer, however, two steps are evident in Fig. 5. The energy gain decreases abruptly after 6 and 12 chlorinations, albeit to a much lesser degree in the latter case. This can be easily understood from the structure of HBC and perchlorinated HBC. The latter is twisted to maximise the Cl–Cl distance.^54^ After 6-fold chlorination, each outer ring of HBC is chlorinated once. Each additional chlorination now encounters a larger steric barrier and the energy gain per chlorination becomes smaller. At a degree of substitution of 12, each ring has been chlorinated twice so that each ring is chlorinated at its positions 1 and 2 while position 3 carries a proton. This symmetry consideration is supported by DFT. By calculating and comparing all twelvefold chlorinated HBC isomers, it was found that this isomer is the most stable. According to this, chlorination 13 to 18 require the overcoming of another steric repulsion, which further reduces the energy gain per chlorination. This finding fits our experimental results. The most intense chlorination degree for the one-pot reaction after one hour is about 10, which is in good agreement with the energetic-geometric reasoning. Furthermore, the system can be forced to perchlorination and the most intense signal can be shifted to 12 if the reaction time is extended to 12 hours. This is also in agreement with the DFT calculations which suggest that higher chlorination requires more energy or time.

It is obvious that our approach does not lead to a single chlorinated species but to a mixture of different chlorinated HBC species. It must be remembered that the reason for the desired edge chlorination is to make the HBC soluble for use in a post-processing application. In these applications, however, the C–Cl bonds are converted back to C–H bonds after processing to recover the desired graphene fragment. Thus, there is no need to force the system to perchlorination using brute force methods. The degree of chlorination only needs to be sufficient to make the compound soluble. Our protocol offers this possibility in a fraction of the time compared to conventional methods, without toxic solvents and elevated temperatures. Nevertheless, the reproducibility of our approach is crucial to provide a viable alternative. To ensure this, we have repeated entry 6 from Table 1 three more times. The average yield was 64% (variation between 56 and 71%). Each experiment was analysed separately with MALDI-TOF and each time showed up to 16-fold chlorination with a similar intensity distribution (Fig. S9†). It should also be noted that in some of the experiments we observed dimer signals of very low intensity compared to the main product.

It is noteworthy that the one-pot synthesis leads to even higher chlorinated HBC than the synthesis starting from planar 3. This can be attributed to the HCl formed during planarization. This increases the pressure in the reaction vessel and contributes *in situ* to the chlorination of the system.

### One-pot bromination of HBC

After we were able to produce 4 with this simple method, we were curious if this approach could be extended to other halogens. Therefore, we advanced our protocol to FeBr_3_ (Table 1, entries 8 and 9). Although soluble products could be obtained, the MALDI-TOF analyses showed that instead of perbrominated 4 a mixture of various brominated oligomers was present. This is exemplified for the reaction after one hour in Fig. 4D, which shows the MALDI-TOF spectra of the soluble and insoluble fractions of this sample. While the soluble fraction shows only traces of 3 and its brominated analogues, it is evident that the insoluble fraction is a mixture of mono-, di- and trimers carrying between two and five bromine atoms. In the case of the 12 hour reaction, even higher oligomers are visible (Fig. S10†). In contrast to FeCl_3_, FeBr_3_ seems to favour oligomerisation over higher halogenation.

## Conclusions

In summary, we have presented a method that enables the edge chlorination of the planar model compound HBC using mechanochemistry and have investigated this method with extensive calculations regarding oligomerisation and chlorination. This method was optimised by a comprehensive evaluation of different chlorination methods, Lewis acids and milling parameters. In contrast to classical methods,^37^ our approach avoids elevated temperatures and the use of toxic solvents such as CCl_4_ in the reaction while an edge-chlorinated nanographene can be presented in a reaction time of only one hour. This approach further requires only FeCl_3_ as a reagent, thus avoiding a combination of multiple chlorination reagents compared to known protocols. While we were even able to carry out the chlorination of HBC metal-free by using oxone and NaCl. Building on this work, we combined the method using FeCl_3_ with the planarization to HBC that we described in previous works.^11,22^ With this one-pot reaction, we were able to halve the number of required reaction steps and prepare edge-chlorinated HBC directly from the dendritic, nonplanar and commercially readily available 5. In the course of this reaction, we were able to obtain 4 in a reaction time of only one hour, while the substitution was driven to completeness by increasing the reaction time and perchlorinated HBC could be successfully prepared. Driven by these results, we finally investigated the edge-bromination of our model compound with FeBr_3_ and found that this favours oligomerization over bromination.

The results described in this work thus open the field to an alternative synthetic pathway to edge-chlorinated nanographenes which not only avoids solvents and other toxic reagents but is also easy to handle and faster than classical methods.

## Conflicts of interest

There are no conflicts to declare.

## Supplementary Material

RA-011-D1RA07679E-s001

## References

[cit1] Novoselov K. S., Geim A. K., Morozov S. V., Jiang D., Zhang Y., Dubonos S. V., Grigorieva I. V., Firsov A. A. (2004). Science.

[cit2] Narita A., Wang X.-Y., Feng X., Müllen K. (2015). Chem. Soc. Rev..

[cit3] Bolotin K. I., Sikes K. J., Jiang Z., Klima M., Fudenberg G., Hone J., Kim P., Stormer H. L. (2008). Solid State Commun..

[cit4] Bolotin K. I., Sikes K. J., Hone J., Stormer H. L., Kim P. (2008). Phys. Rev. Lett..

[cit5] Dutta S., Pati S. K. (2010). J. Mater. Chem..

[cit6] Ponomarenko L. A., Schedin F., Katsnelson M. I., Yang R., Hill E. W., Novoselov K. S., Geim A. K. (2008). Science.

[cit7] Kosynkin D. V., Higginbotham A. L., Alexander S., Lomeda J. R., Dimiev A., Katherine Price B., Tour J. M. (2009). Nature.

[cit8] Leclerc M. and Morin J.-F., Synthetic Methods for Conjugated Polymers and Carbon Materials, Wiley-VCH Verlag GmbH & Co. KGaA, Weinheim, Germany, 2017

[cit9] Scholl R., Mansfeld J. (1910). Ber. Dtsch. Chem. Ges..

[cit10] Navakouski M., Zhylitskaya H., Chmielewski P. J., Żyła-Karwowska M., Stępień M. (2020). J. Org. Chem..

[cit11] Grätz S., Beyer D., Tkachova V., Hellmann S., Berger R., Feng X., Borchardt L. (2018). Chem. Commun..

[cit12] James S. L., Adams C. J., Bolm C., Braga D., Collier P., Friščić T., Grepioni F., Harris K. D. M., Hyett G., Jones W., Krebs A., Mack J., Maini L., Orpen A. G., Parkin I. P., Shearouse W. C., Steed J. W., Waddell D. C. (2012). Chem. Soc. Rev..

[cit13] Hernández J. G., Bolm C. (2017). J. Org. Chem..

[cit14] James S. L., Friščić T. (2013). Chem. Soc. Rev..

[cit15] Do J.-L., Friščić T. (2017). Synlett.

[cit16] Tetzlaff D., Pellumbi K., Baier D. M., Hoof L., Shastry Barkur H., Smialkowski M., Amin H. M. A., Grätz S., Siegmund D., Borchardt L., Apfel U.-P. (2020). Chem. Sci..

[cit17] Leistenschneider D., Jäckel N., Hippauf F., Presser V., Borchardt L. (2017). Beilstein J. Org. Chem..

[cit18] Schneidermann C., Jäckel N., Oswald S., Giebeler L., Presser V., Borchardt L. (2017). ChemSusChem.

[cit19] Shi W., Yu J., Jiang Z., Shao Q., Su W. (2017). Beilstein J. Org. Chem..

[cit20] Yuan W., Friscić T., Apperley D., James S. L. (2010). Angew. Chem., Int. Ed. Engl..

[cit21] Užarević K., Wang T. C., Moon S.-Y., Fidelli A. M., Hupp J. T., Farha O. K., Friščić T. (2016). Chem. Commun..

[cit22] Krusenbaum A., Grätz S., Bimmermann S., Hutsch S., Borchardt L. (2020). RSC Adv..

[cit23] Sim Y., Shi Y. X., Ganguly R., Li Y., García F. (2017). Chem.–Eur. J..

[cit24] Tan D., García F. (2019). Chem. Soc. Rev..

[cit25] Rightmire N. R., Hanusa T. P. (2016). Dalton Trans..

[cit26] Hermann G. N., Becker P., Bolm C. (2016). Angew. Chem., Int. Ed. Engl..

[cit27] Stolle A., Szuppa T., Leonhardt S. E. S., Ondruschka B. (2011). Chem. Soc. Rev..

[cit28] Bernhardt F., Trotzki R., Szuppa T., Stolle A., Ondruschka B. (2010). Beilstein J. Org. Chem..

[cit29] Komatsu K., Fujiwara K., Murata Y. (2000). Chem. Commun..

[cit30] Komatsu K., Wang G.-W., Murata Y., Tanaka T., Fujiwara K., Yamamoto K., Saunders M. (1998). J. Org. Chem..

[cit31] Grätz S., Borchardt L. (2016). RSC Adv..

[cit32] Báti G., Csókás D., Yong T., Tam S. M., Shi R. R. S., Webster R. D., Pápai I., García F., Stuparu M. C. (2020). Angew. Chem., Int. Ed. Engl..

[cit33] Yong T., Báti G., García F., Stuparu M. C. (2021). Nat. Commun..

[cit34] Bratz L. T., Von Niementowski S. (1919). Ber. Dtsch. Chem. Ges. (A and B Ser.).

[cit35] Sahoo A. K., Nakamura Y., Aratani N., Kim K. S., Noh S. B., Shinokubo H., Kim D., Osuka A. (2006). Org. Lett..

[cit36] Lewtak J. P., Gryko D., Bao D., Sebai E., Vakuliuk O., Ścigaj M., Gryko D. T. (2011). Org. Biomol. Chem..

[cit37] Tan Y.-Z., Yang B., Parvez K., Narita A., Osella S., Beljonne D., Feng X., Müllen K. (2013). Nat. Commun..

[cit38] Schmidt R., Stolle A., Ondruschka B. (2012). Green Chem..

[cit39] TURBOMOLE V7.3 2018, a development of University of Karlsruhe and Forschungszentrum Karlsruhe GmbH, TURBOMOLE GmbH, 1989–2007, since 2007; available from http://www.turbomole.com/

[cit40] Becke A. D. (1998). J. Chem. Phys..

[cit41] Lee C., Yang W., Parr R. G. (1988). Phys. Rev. B: Condens. Matter Mater. Phys..

[cit42] Grimme S., Antony J., Ehrlich S., Krieg H. (2010). J. Chem. Phys..

[cit43] Grimme S., Ehrlich S., Goerigk L. (2011). J. Comput. Chem..

[cit44] Weigend F., Ahlrichs R. (2005). Phys. Chem. Chem. Phys..

[cit45] Oliver T., Ahlrichs R. (1998). J. Chem. Phys..

[cit46] Eichkorn K., Treutler O., Öhm H., Häser M., Ahlrichs R. (1995). Chem. Phys. Lett..

[cit47] Eichkorn K., Weigend F., Treutler O., Ahlrichs R. (1997). Theor. Chem. Acc..

[cit48] Plimpton S. (1995). J. Comput. Phys..

[cit49] Bannwarth C., Caldeweyher E., Ehlert S., Hansen A., Pracht P., Seibert J., Spicher S., Grimme S. (2021). Wiley Interdiscip. Rev.: Comput. Mol. Sci..

[cit50] Humphrey W., Dalke A., Schulten K. (1996). J. Mol. Graphics.

[cit51] Stone J. , MA thesis, Computer Science Department, University of Missouri-Rolla, 1998

[cit52] Bannwarth C., Ehlert S., Grimme S. (2019). J. Chem. Theory Comput..

[cit53] King B. T., Kroulík J., Robertson C. R., Rempala P., Hilton C. L., Korinek J. D., Gortari L. M. (2007). J. Org. Chem..

[cit54] Maghsoumi A., Narita A., Dong R., Feng X., Castiglioni C., Müllen K., Tommasini M. (2016). Phys. Chem. Chem. Phys..

