# Supplementary material for: Beyond the Scholl reaction – one-step planarization and edge chlorination of nanographenes by mechanochemistry
Source: RSC Adv. 2021 Nov 25;11(60):38026–32. doi: 10.1039/d1ra07679e (PMC9044044; doi:10.1039/d1ra07679e)
Supplement: RA-011-D1RA07679E-s001 [file RA-011-D1RA07679E-s001.pdf]

Electronic Supplementary Material (ESI) for RSC Advances.
This journal is © The Royal Society of Chemistry 2021

Beyond the Scholl Reaction – One-Step Planarization and Edge-Chlorination of Nanographenes by Mechanochemistry

Daniel M. Baier[a], Sven Grätz[a], Babak Farhadi Jahromi[b], Sarah Hellmann[c], Konrad Bergheim[a], Wilm Pickhardt[a], Rochus Schmid[b] and Lars Borchardt*[a]

a Mechanochemistry Group, Faculty for Chemistry and Biochemistry, Ruhr-Universität Bochum, Universitätsstraße 150, 44780 Bochum, Germany.

b Computational Materials Chemistry group, Faculty for Chemistry and Biochemistry, Ruhr-Universität Bochum,
Universitätsstraße 150, 44780 Bochum, Germany.

c Professur für Anorganische Chemie I, Technische Universität Dresden, Bergstraße 66, D-01069 Dresden, Germany.

Table of Contents

1 General methods ... 2
2 Materials ... 2
3 Summary of the results with alternative Lewis acids and chlorination methods ... 2
4 Synthetic Procedures ... 3
4.1 Synthesis of nanographenes ... 3
4.2 General Mechanochemical edge-chlorination procedure ... 3
4.2.1 Chlorination of HBC ... 3
4.2.2 Chlorination of Triphenylene ... 5
4.3 One-step planarization and edge-chlorination procedure ... 5
5 Characterization ... 6
5.1 IR-Spectroscopy ... 6
5.2 X-Ray Diffraction ... 7
5.3 MALDI-TOF ... 8
5.4 UV/Vis ... 12
6 Photos of the Toluene Solutions of the One-Pot Reactions with $FeBr_3$ ... 13
7 Computational Study ... 13
8 Author contributions ... 19
9 References ... 19

1 General methods

Ball mill syntheses were carried out in a planetary ball mill (*Fritsch* Pulverisette 7 premium line) at a rotational speed of 800 rpm. The reactions were performed in a 20 mL milling vessel made of ZrO_2 with 10 milling balls (ZrO_2, 10 mm in diameter) if not stated otherwise. Additionally, a *Retsch* EMAX high-energy ball mill with a 50 ml ZrO_2 milling vessel and 25 milling balls (ZrO_2, 10 mm in diameter) with a frequency of 1200 rpm and 60 minutes was used.

Bulking material: A bulking agent is an inert additive to the reaction. The size of the milling vessel (20 or 50 mL in the present work) requires a minimum amount of reactants in the milling vessel (ball-to-powder ratio), otherwise the milling balls will collide with each other without transferring the collision energy to the reactants, which causes abrasion of the milling material. We used NaCl as an inert bulking material. This method was first described by Konnert et al.[1] Since we used a total mass of 2 g in the 20 ml milling vessel, we added the amount of NaCl that was necessary to reach this mass. As an alternative we could have also increased the amount of reactants to reach the required mass.

2 Materials

Aluminium chloride (Sigma Aldrich), Chloroform (VWR, 99.3%), Ethanol (VWR, 99.9%), Hexaphenylbenzene (TCI), Iodine Monochloride (ABCR, no purity stated), Iron(III) bromide (anhydrous; Alfa Aesar, >98%), Iron(III) chloride (anhydrous; ABCR, >98%), Methanol (VWR, 99.9%), Molybdenum(V) chloride (anhydrous; ABCR, 99.6%), Copper(II) chloride (Sigma Aldrich, 99%), Oxone (Sigma Aldrich, no purity stated), N-chlorosuccinimide (Sigma Aldrich, 98%), sulfuric acid (Fisher scientific, >=95%), o-Terphenyl (Sigmal Aldrich, 99%), Toluene (VWR, 99.9%), Trichloroisocyanuric acid (TCI, 95%), Triphenylene (Alfa Aesar, 98%) and Sodium chloride (Güssig, >98%) were used as received.

ZrO_2 (Type ZY-S) milling balls (diameter: 10 mm; average weight: 3.19 ± 0.05 g) were purchased from Sigmund Lindner GmbH.

3 Summary of the results with alternative Lewis acids and chlorination methods

Tab. S1. Reaction conditions and yields of the chlorination of hexabenzocoronene (**3**) using different Lewis acids (S1-S4) and using different chlorination methods (S5-S8). NCS stands for *N*-chlorosuccinimide; Reaction conditions if not stated otherwise: 800 rpm, 10x10 mm balls, ZrO_2, 60 min, 2g of powder, 5 wt% substrate.

Entry	Method	Mill	Time (min.)	Sol. Fraction[a] (%)	Yield[b] (%)
S1	$CuCl_2$	P7	60	8	5
S2	$MoCl_5$	P7	60	11	15
S3	$AlCl_3$	P7	60	1	0.5
S4	$AlCl_3$	P7	720	1	0.5
S5	ICl, AlC_{l3}	P7	60	33	21
S6[c]	$C_3Cl_3N_3O_3$, $AlCl_3$, H_2SO_4	P7	120	24	17
S7	NCS, $FeCl_3$	P7	60	37	20
S8	Oxone, NaCl	P7	60	83	43

[a] After the reaction, the crude reaction mixture is taken up in water, filtered and washed with MeOH and EtOH. The residue obtained is dried overnight at 80 °C and then extracted with $CHCl_3$. This is the soluble fraction. [b] yield calculated for the soluble fraction in regard to perchlorinated Nanographenes, [c] only 2.5 wt% substrate.

4 Synthetic Procedures

4.1 Synthesis of nanographenes

Hexa-peri-hexabenzocoronene [(HBC), $C_{42}H_{18}$] was synthesized according to the literature.[2]

4.2 General Mechanochemical edge-chlorination procedure

4.2.1 Chlorination of HBC

Method A: Metal chloride

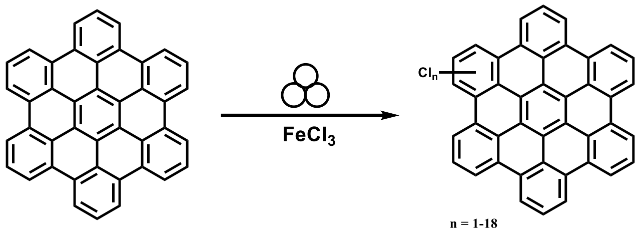

1 2

In a typical synthesis, 0.1 g HBC **1** (0.192 mmol) and an excess of 1.9 g metal chloride (e.g. iron(III) chloride (11.75 mmol, 61 eq.)) were transferred into a 20 mL zirconium oxide grinding jar with ten zirconium oxide 10 mm-diameter grinding balls (3.19 g each). The mixture was then milled for 60 min at 800 rpm in a *Fritsch* Pulverisette 7 premium line planetary ball mill. After the reaction, the grinding jar was opened and the reaction mixture was poured into water. The crude product was consequently washed with water, methanol and ethanol. The soluble fraction was extracted with $CHCl_3$ which was consequently evaporated and the solid was dried at 80°C. Chlorinated HBC **2** was obtained as a dark red solid (107.5 mg, yield: 49 %). MALDI-TOF **(Fig. 3)** (TCNQ): 937.14, calc. 1142.60.

Other metal chlorides used with the same mass ratio: $CuCl_2$, $AlCl_3$, $MoCl_5$

Method B: ICl, $AlCl_3$

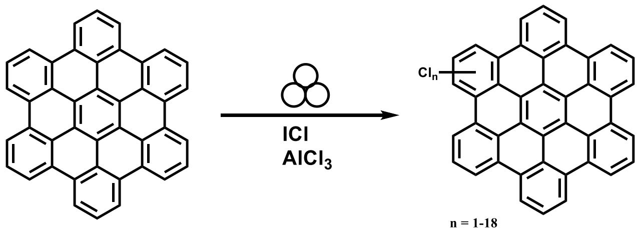

1 2

In a typical synthesis, 0.03 g HBC **7** (0.057 mmol), an excess of 0.563 g aluminum chloride (4.133 mmol, 72 eq.), 1.62 g iodine monochloride (0.5 ml, 9.98 mmol, 174 eq.) and 1.4 g of sodium chloride as bulking material were transferred into a 20 mL zirconium oxide grinding jar with ten zirconium oxide 10 mm-diameter grinding balls (3.19 g each). The mixture was then milled for 60 min at 800 rpm in a *Fritsch* Pulverisette 7 premium line planetary ball mill. After the reaction, the grinding jar was opened and the reaction mixture was poured into water. The crude product was consequently washed with water, methanol and ethanol. The soluble fraction was extracted with $CHCl_3$ which was consequently evaporated and the solid was dried at 80°C. Chlorinated HBC **2** was obtained as a dark red solid (13.7 mg, yield: 21 %). MALDI-TOF **(Fig. S5)** (TCNQ): 898.99, calc. 1142.60.

Method C: $C_3Cl_3N_3O_3$, $AlCl_3$, H_2SO_4

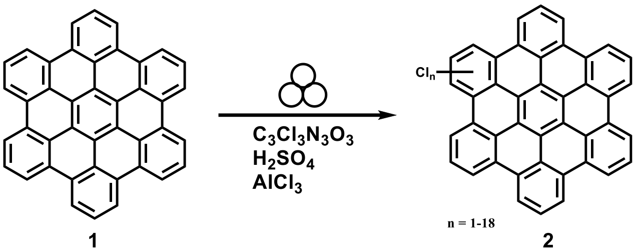

In a typical synthesis, 0.05 g HBC **1** (0.096 mmol), an excess of 1.855 g aluminum chloride (13.91 mmol, 145 eq.), 0.290 g trichloroisocyanuric acid (1.25 mmol, 13 eq.) and 1 mL of concentrated sulphuric acid were transferred into a 20 mL zirconium oxide grinding jar with ten zirconium oxide 10 mm-diameter grinding balls (3.19 g each). The mixture was then milled for 60 min at 800 rpm in a *Fritsch* Pulverisette 7 premium line planetary ball mill. After the reaction, the grinding jar was opened and the reaction mixture was poured into water. The crude product was consequently washed with water, methanol and ethanol. The soluble fraction was extracted with $CHCl_3$ which was consequently evaporated and the solid was dried at 80°C. Chlorinated HBC **2** was obtained as a dark red solid (19 mg, yield: 17 %). MALDI-TOF **(Fig. S6)** (TCNQ): 898.21, calc. 1142.60.

Method D: NCS, $FeCl_3$

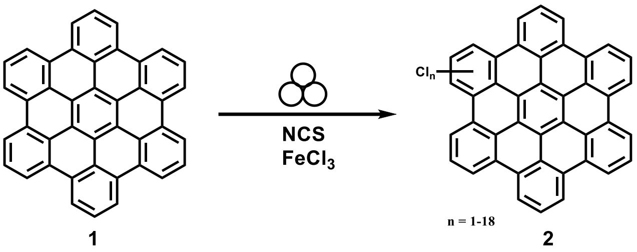

In a typical synthesis, 0.03 g HBC **1** (0.057 mmol), an excess of 0.335 g iron(III) chloride (2.067 mmol, 36 eq.), 0.276 g N-chlorosuccinimide (2.067 mmol, 36 eq.) and 1.4 g of sodium chloride as bulking material were transferred into a 20 mL zirconium oxide grinding jar with ten zirconium oxide 10 mm-diameter grinding balls (3.19 g each). The mixture was then milled for 60 min at 800 rpm in a *Fritsch* Pulverisette 7 premium line planetary ball mill. After the reaction, the grinding jar was opened and the reaction mixture was poured into water. The crude product was consequently washed with water, methanol and ethanol. The soluble fraction was extracted with $CHCl_3$ which was consequently evaporated and the solid was dried at 80°C. Chlorinated HBC **2** was obtained as a dark red solid (13.0 mg, yield: 20 %). MALDI-TOF **(Fig. S7)** (TCNQ): 522.53, calc. 1142.60.

Method E: Oxone

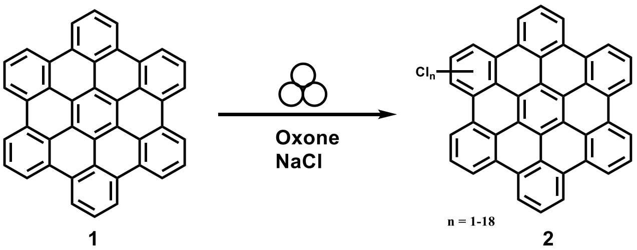

In a typical synthesis, 0.1 g HBC **1** (0.192 mmol), an excess of 1.413 g Oxone (KHSO5 · 0.5KHSO4 · 0.5K2SO4) (4.596 mmol, 24 eq.) and 0.487 g sodium chloride (8.338 mmol, 44 eq.) were transferred into a 20 mL zirconium oxide grinding jar with ten zirconium oxide 10 mm-diameter grinding balls (3.19 g each). The mixture was then milled for 60 min at 800 rpm in a *Fritsch* Pulverisette 7 premium line planetary ball mill. After the reaction, the grinding jar was opened and the reaction mixture was poured into water. The crude product was consequently washed with water, methanol and ethanol. The soluble fraction was extracted with $CHCl_3$ which was consequently evaporated and the solid was dried at 80°C. Chlorinated HBC **2** was obtained as a dark red solid (94.3 mg, yield: 43 %). MALDI-TOF **(Fig. S8)** (TCNQ): 868.51, calc. 1142.60.

4.2.2 Chlorination of Triphenylene

Method A: Metal chloride

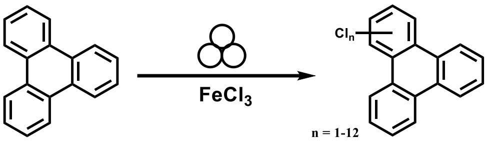

3 **4**

0.1 g Triphenylene **3** (0.438 mmol) and an excess of 1.9 g iron(III) chloride (11.75 mmol, 27 eq.) were transferred into a 20 mL zirconium oxide grinding jar with ten zirconium oxide 10 mm-diameter grinding balls (3.19 g each). The mixture was then milled for 60 min at 800 rpm in a *Fritsch* Pulverisette 7 premium line planetary ball mill. After the reaction, the grinding jar was opened and the reaction mixture was poured into water. The crude product was consequently washed with water, methanol and ethanol. The soluble fraction was extracted with $CHCl_3$ which was consequently evaporated and the solid was dried at 80°C. Chlorinated Triphenylene **4** was obtained as a brown solid (146.6 mg, yield: 52 %). MALDI-TOF **(Fig. 2)** (TCNQ): 543.32, calc. 641.63.

4.3 One-step planarization and edge-chlorination procedure

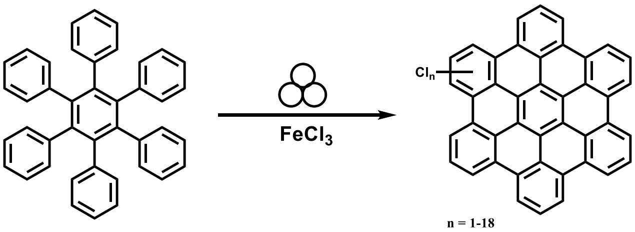

5 **2**

In a typical synthesis, 0.1 g hexaphenylbenzene **5** (0.187 mmol) and 2.18 g iron(III) chloride (13.44 mmol, 72 eq.) were transferred into a 20 mL zirconium oxide grinding jar with ten zirconium oxide 10 mm-diameter grinding balls (3.19 g each). The mixture was then milled for 60 min at 800 rpm in a *Fritsch* Pulverisette 7 premium line planetary ball mill. After the reaction, the grinding jar was opened and the reaction mixture was poured into water. The crude product was consequently washed with water, methanol and ethanol. The soluble fraction was extracted with $CHCl_3$ which was consequently evaporated and the solid was dried at 80°C. Chlorinated HBC **2** was obtained as a dark red solid (89 mg, yield: 42%). MALDI-TOF **(Fig. 4C)** (TCNQ): 763.15, calc. 1142.60.

In the case of attempted bromination, 3.98g iron(III) bromide (13.47, 72 eq.) were used.

5 Characterization

5.1 IR-Spectroscopy

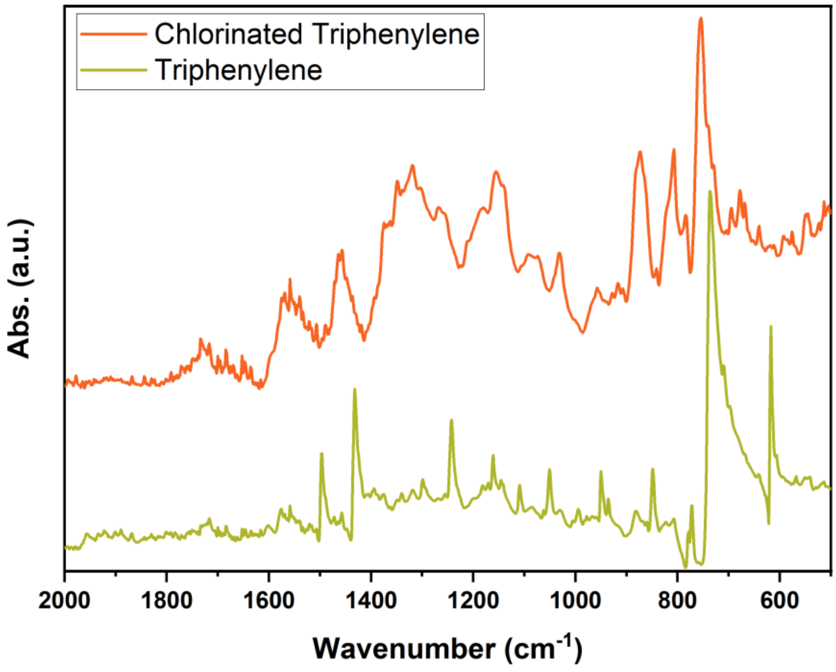

Fig. S1. IR-spectra of the Triphenylene reference and its chlorination by $FeCl_3$ (Table 1, entry 1).

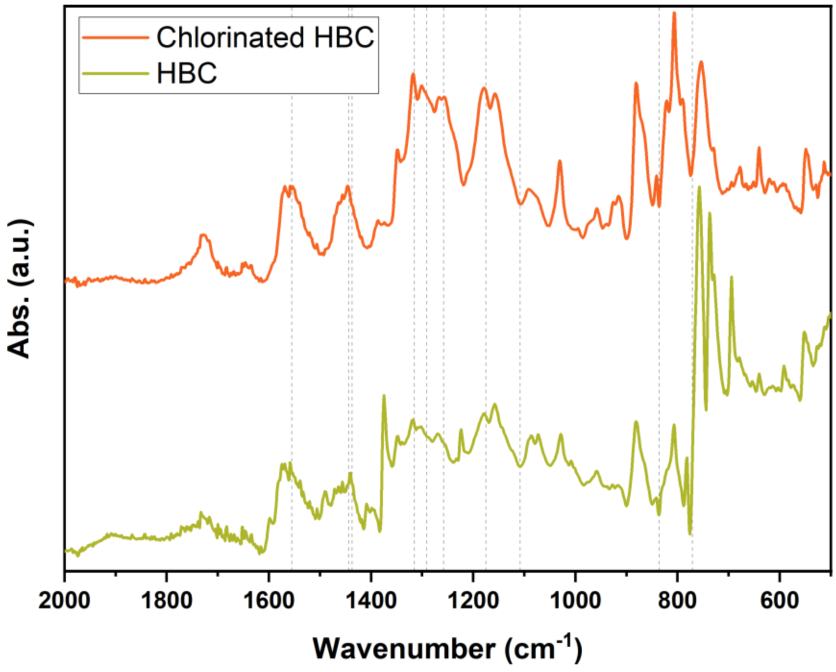

Fig. S2. IR-spectra of HBC and its chlorination by $FeCl_3$ (Table 1, entry 2). Grey dotted lines belong to the literature values of perchlorinated HBC.[3]

5.2 X-Ray Diffraction

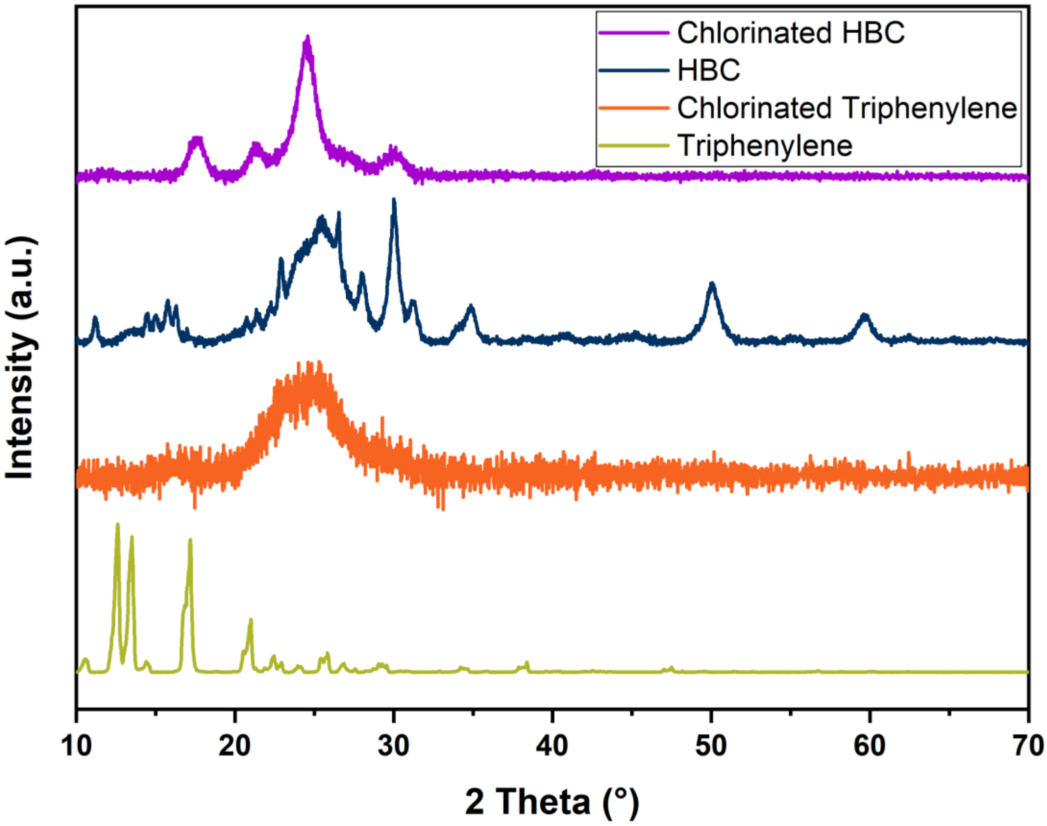

Fig. S3. X-Ray diffractograms of the triphenylene reference, chlorinated triphenylene (Table 1, entry 1), HBC and of chlorinated HC (Table 1, entry 2).

5.3 MALDI-TOF

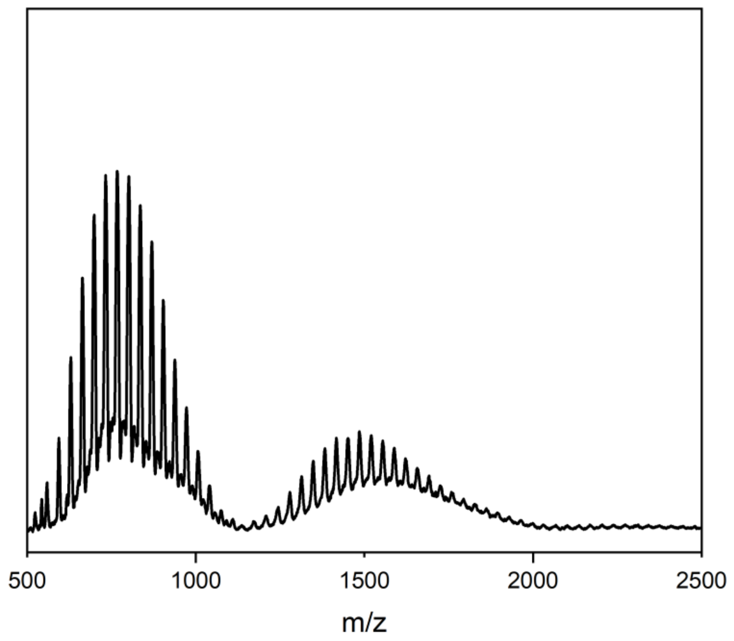

Fig. S4. MALDI-TOF spectrum of the chlorinated HBC synthesized with $FeCl_3$ in a EMAX high-energy ball mill (Table 1, entry 5).

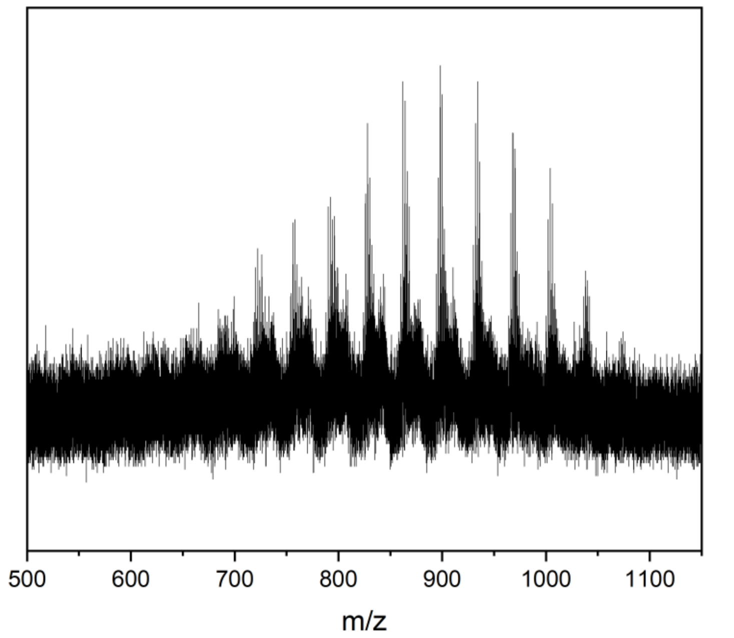

Fig. S5. MALDI-TOF spectrum of the chlorinated HBC synthesized with ICl_3 and $AlCl_3$ (Table S1, entry S5).

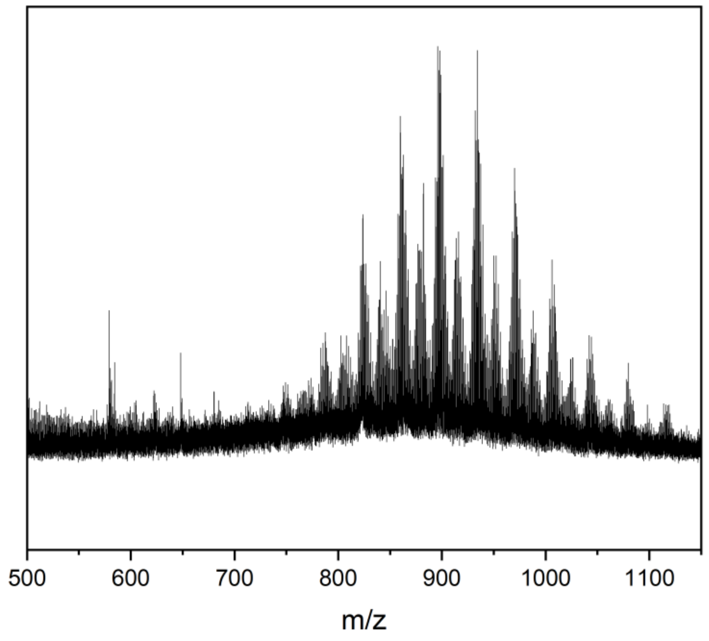

Fig. S6. MALDI-TOF spectrum of the chlorinated HBC synthesized with $C_3Cl_3N_3O_3$, $AlCl_3$ and H_2SO_4 (Table S1, entry S6).

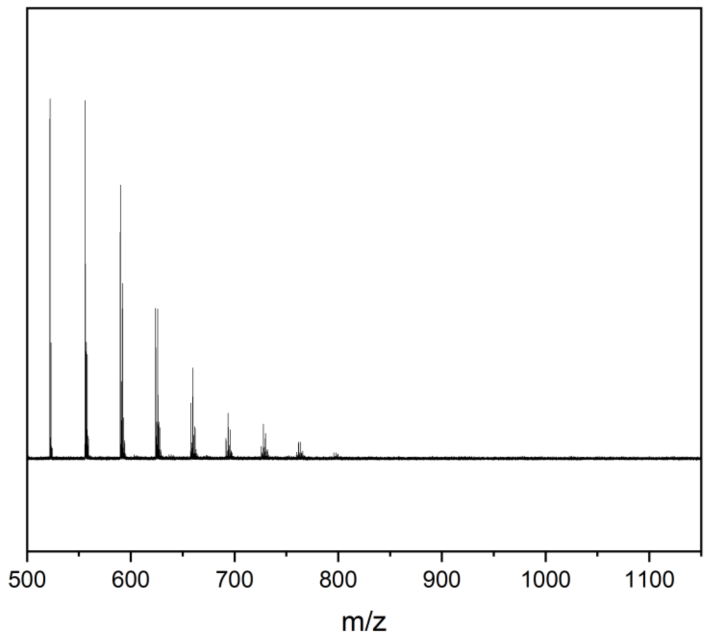

Fig. S7. MALDI-TOF spectrum of the chlorinated HBC synthesized with NCS and $FeCl_3$ (Table S1, entry S7).

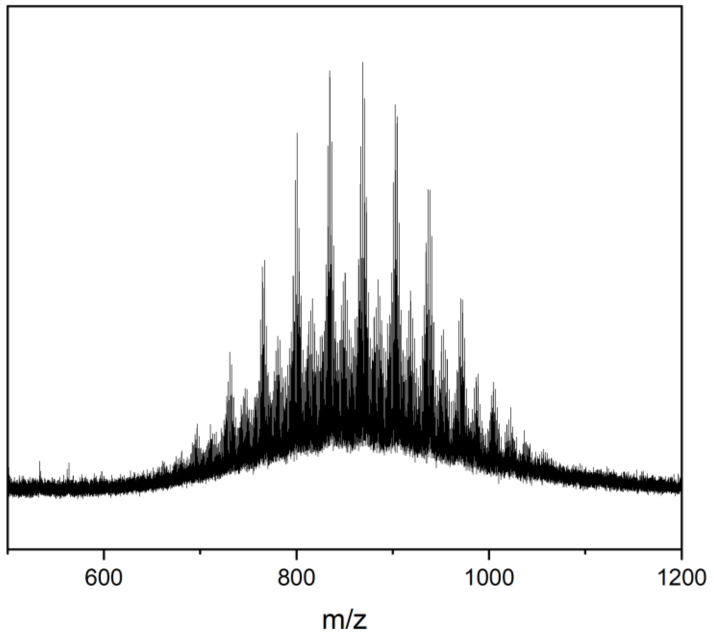

Fig. S8. MALDI-TOF spectrum of the chlorinated HBC synthesized with Oxone (Table S1, entry S8).

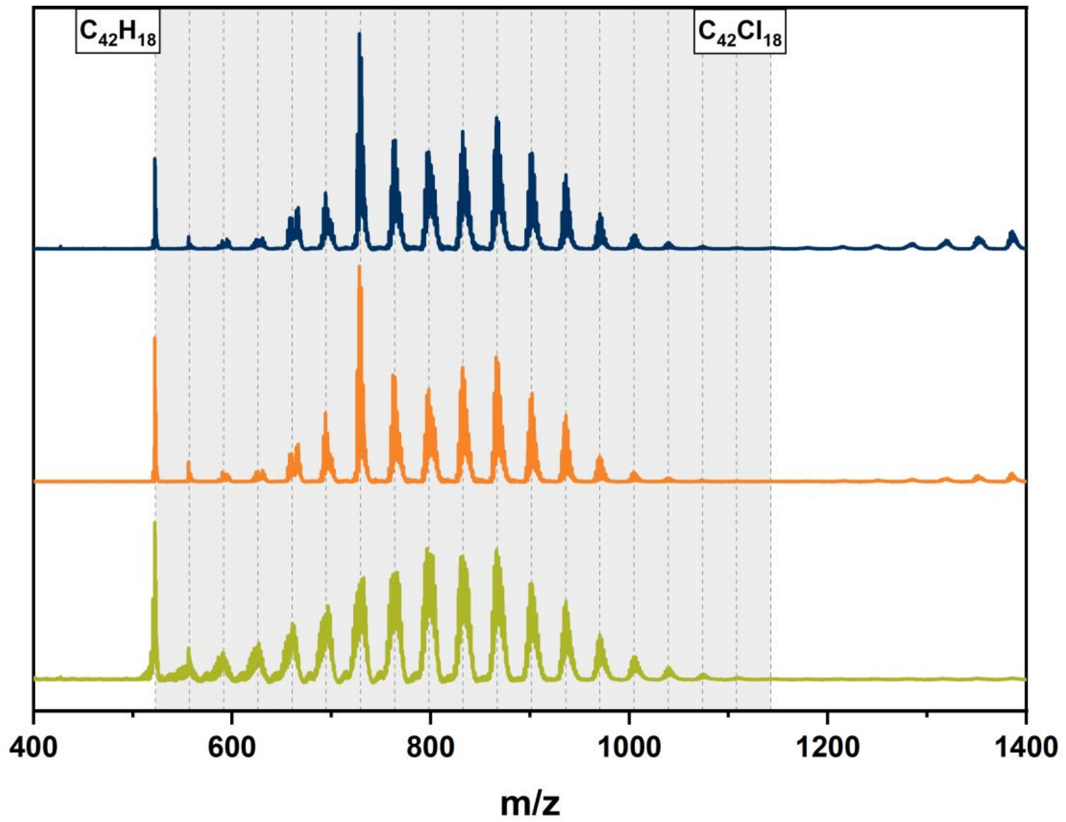

Fig. S9. Reproduction of entry 6 in Table 1: MALDI-TOF spectra of chlorinated HBC synthesised with $FeCl_3$ after 1h (three separate reactions).

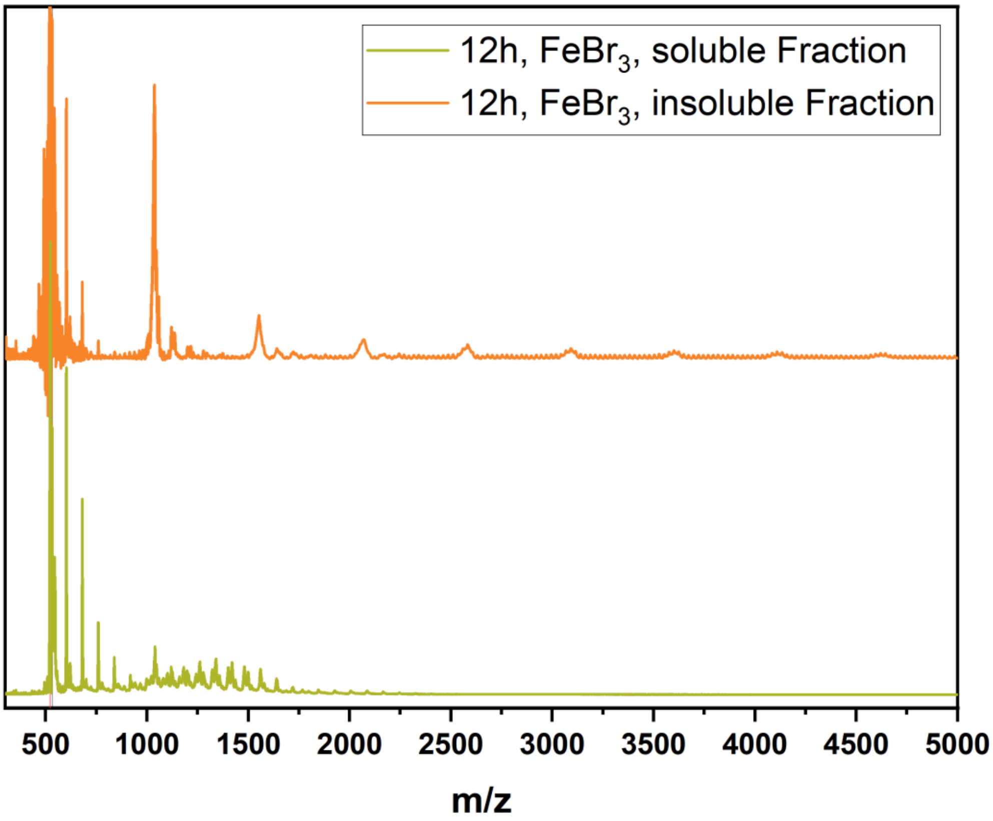

Fig. S10. **MALDI-TOF spectra of the soluble and insoluble fraction of the brominated HBC from the one-pot reaction with $FeBr_3$ after 12 hours (Table 1, entry 9).**

5.4 UV/Vis

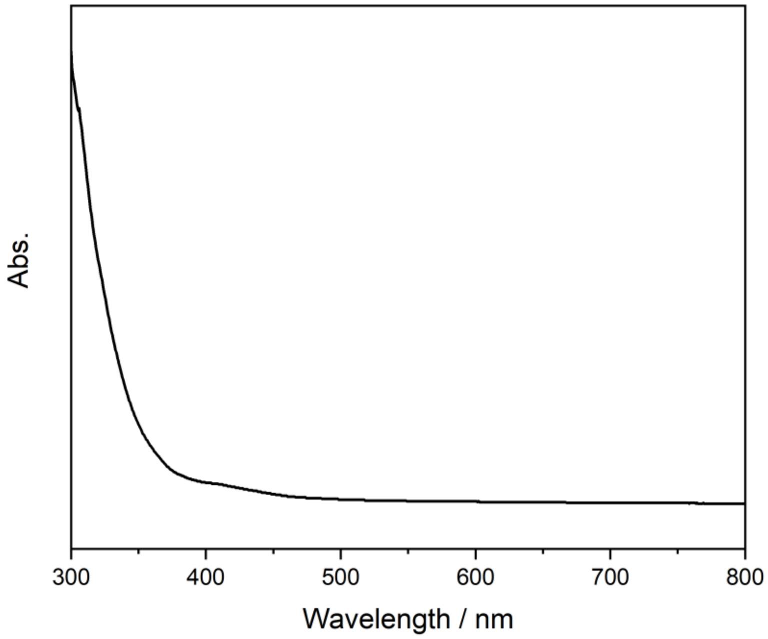

Fig. S11. UV/Vis spectrum of the chlorinated Triphenylene dissolved in toluene synthesized with $FeCl_3$ (Table 1, entry 1).

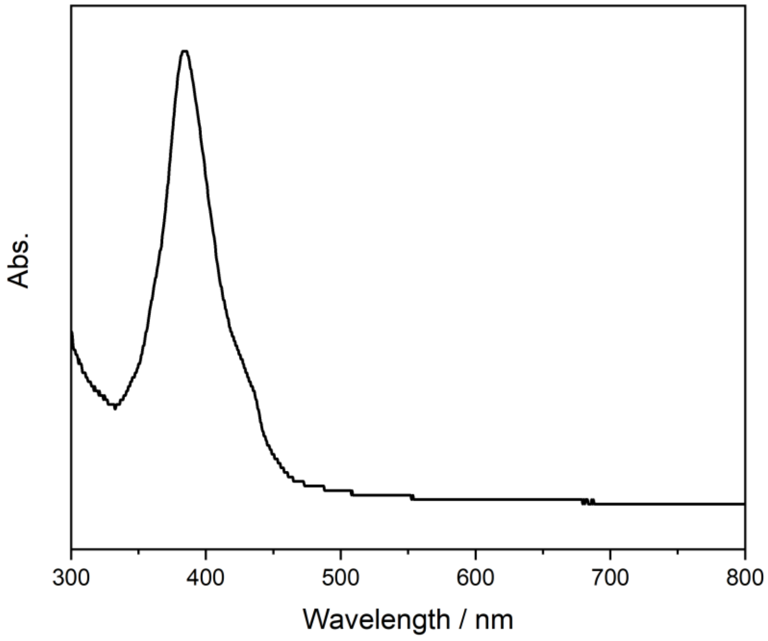

Fig. S12. UV/Vis spectrum of the chlorinated HBC dissolved in toluene synthesized with $FeCl_3$ in a EMAX high-energy ball mill (Table 1, entry 5).

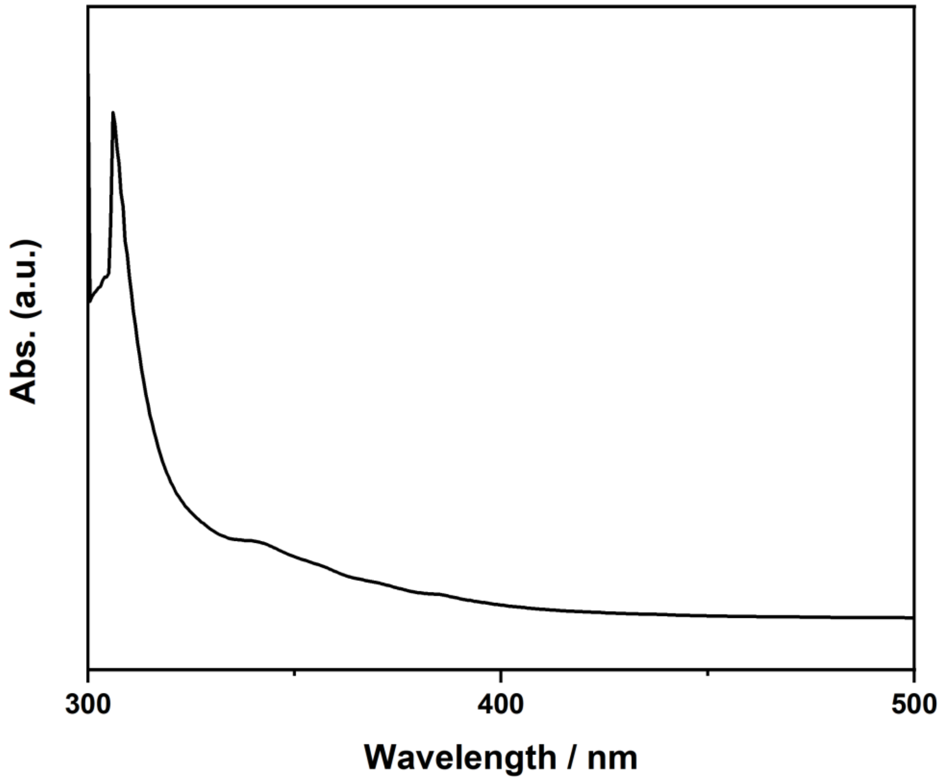

Fig. S13. UV/Vis spectrum of the brominated HBC dissolved in toluene from the one-pot reaction synthesized with $FeBr_3$ after one hour (Table 1, entry 8).

6 Photos of the Toluene Solutions of the One-Pot Reactions with $FeBr_3$

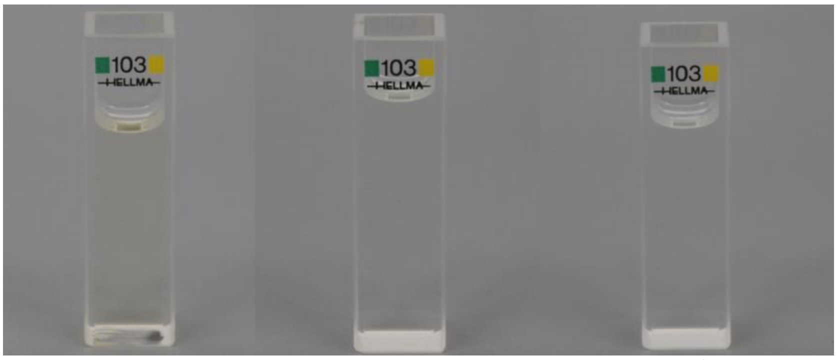

Fig. S14. Photos of the toluene solutions of Table 1, entries 8 and 9 and of the hexaphenylbenzene reference (photos according to the enumeration).

7 Computational Study

For the determination of the energetically most stable oligomer structures, initial geometries for all possible oligomers were generated using a simple algorithm that functionalizes an arbitrary number of initial structures, in this case a single triphenylene structure, with an arbitrary number of functional groups, in this case 1- and 2-triphenylenyl, at every possible functionalization site, where every unique structure it generates is added to the pool of structures to be functionalized, until a certain stopping criterion is met. The stopping criterion that was used here, was the generation of the first pentamer, which was then discarded. The generated molecules are tested for uniqueness by registering their respective canonical SMILES as produced by the Open Babel library[4] and comparing with the already registered ones. The recursive nature of the algorithm ensures the generation of all possible oligomers. To increase the chance that the respective global minima are found in the geometry optimizations of the

generated initial structures, rather than energetically less preferable conformers, each unique structure is cloned and rotated around the C-C-C-C dihedral angle, where the two central carbon atoms form the new bond between input structure and functional group, and the rotated structure is also added to the structure pool. Due to the nature of the algorithm, further geometries are only derived from the initially found unique structure and not from the rotated clone, which limits the effectiveness of this procedure to some degree but also the total number of initial geometries which need to be optimized. Unique structures (or rotated clones of these) with unreasonably close atom distances such as in the case of overlapping molecule segments are automatically discarded by the algorithm. In total, six dimer structures for three unique dimer SMILES, 87 trimer structures for 46 unique trimer SMILES and 2069 tetramer structures for 1122 unique tetramer SMILES were found by the algorithm.

Facing the large number of geometries to be optimized, a strategy was chosen that maximizes computational efficiency without sacrificing much of the accuracy of the results. Rather than optimizing all initial oligomer structures at the desired level of theory for the final results, they were optimized with the much faster extended tight-binding method GFN2-xTB, developed by Grimme *et al.*[5], and were ranked according to their total molecular energies at the optimum geometries predicted by this method. A selection of the most favorable structures according to this ranking then provided the initial geometries for the final DFT calculations, except for the dimer case, where all six structures were optimized using both methods, serving as a small test study to verify the validity of this procedure. The relative molecular energies of the six dimer structures computed via both methods and the corresponding optimized geometries obtained from DFT calculations are displayed in **Fig. S15**. The geometries obtained from the GFN2-xTB method are reasonably close to the DFT-optimized ones, as seen in **Fig. S16**.

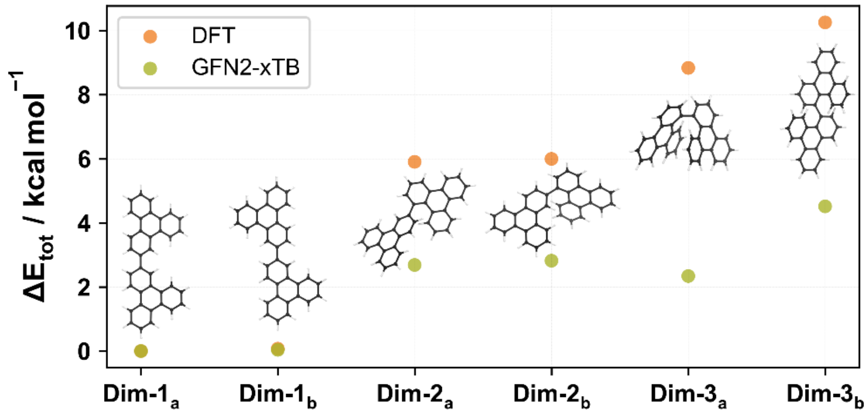

Fig. S15. **Relative molecular energies of the six considered dimer structures, according to GFN2-xTB and DFT, as well as the respective DFT-optimized geometries.**

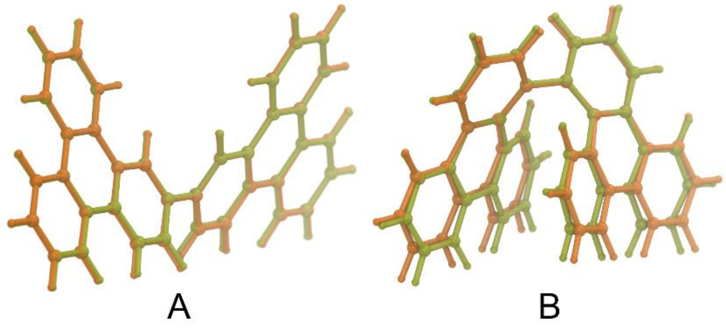

Fig. S16. **Overlayed optimized geometries of A: Dim-1a and B: Dim-3a, obtained from GFN2-xTB (orange) and DFT (blue) calculations.**

The six optimized dimer geometries can be identified as conformer pairs of each of the three possible constitutional isomers. Regarding the two used methods, GFN2-xTB generally underestimates the energy differences between the six dimer geometries, in other words, it overstabilizes the less preferred geometries compared to DFT. While **Dim-1** is predicted by both methods to be the thermodynamically most stable isomer, **Dim-1$_a$** being slightly preferred over **Dim-1$_b$** by both methods, there is a discrepancy regarding the stability of **Dim-3$_a$**, which the GFN2-xTB method predicts to be much more stable than the DFT calculations do, up to the point where it is preferred over both conformers of **Dim-2,** whereas both methods are in accord about the least preferable geometry of **Dim-3$_b$**. Taking these observations into account, GFN2-xTB, while its results quantitatively not agreeing with those computed via DFT, can be considered a valid method for pre-optimizing the initial geometries generated by the above described algorithm and pre-filtering the geometries that are to be further optimized using DFT, since it allows for a qualitative stability ranking of the considered structures.

To remedy the fact, that, with increasing structural complexity, the chance of optimizing towards local minima increases, the pool of trimer structures to be optimized was further supplied with the set of trimer structures that is obtained from running the above-mentioned algorithm with **Dim-1$_a$** as the single starting structure, ignoring whether this results in any duplicate calculations. The twelve lowest-energy trimer geometries according to GFN2-xTB were then further optimized using DFT. Out of the resulting structures, the four best are displayed in **Fig. S17**. Analogously, for the tetramer structures, direct descendants of **Dim-1$_a$** and **Tri-1**, as generated by the above-described algorithm, were added to the pool of structures to be pre-optimized using GFN2-xTB. This pool was further supplied with the set of tetramer structures that are directly generated by functionalizing **Dim-1$_a$** with all fragments that can be derived from **Dim-1$_a$** itself by abstracting a single hydrogen atom. Here, the 16 most favorable geometries, as predicted by GFN2-xTB, were reoptimized using DFT, the four best out of the resulting structures being displayed in **Fig. S18**.

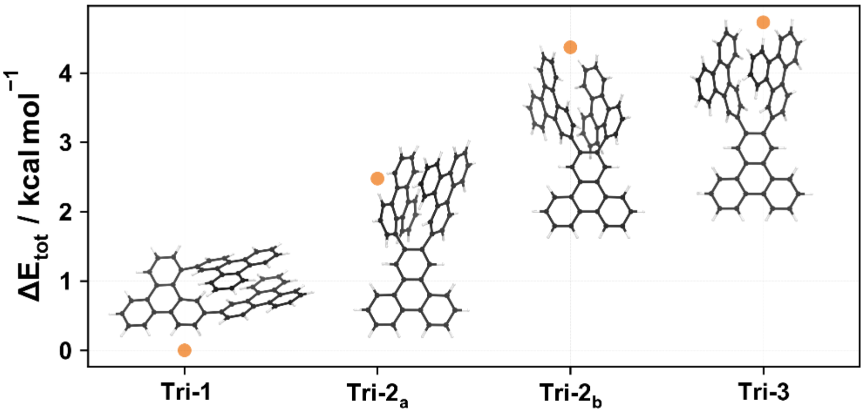

Fig. S17. **Relative molecular energies of the four most preferable trimer structures according to DFT, as well as the respective DFT-optimized geometries.**

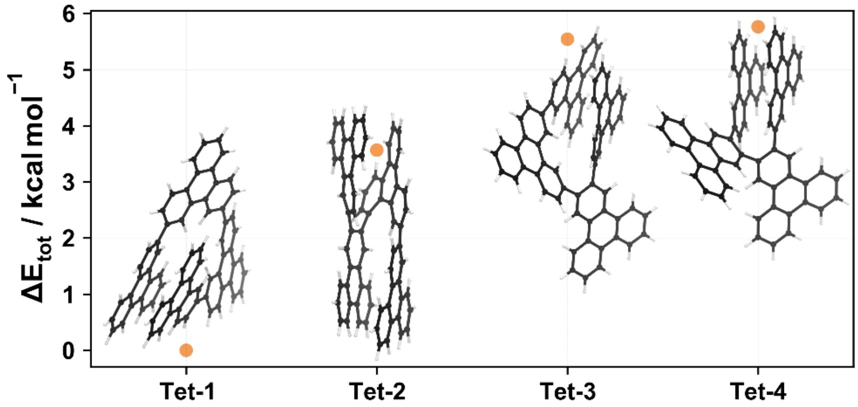

Fig. S18. **Relative molecular energies of the four most preferable tetramer structures according to DFT, as well as the respective DFT-optimized geometries.**

For the computation of the energetically most favorable chlorinated isomers of **Dim-1$_a$** and HBC a modified version of the above-described algorithm was used, where instead of replacing hydrogen atoms by functional groups, these are simply substituted with chlorine atoms. While for HBC, the optimization of all 22284 unique chlorinated isomers (including non-chlorinated HBC) with GFN2-xTB was still computationally feasible, this was not the case for **Dim-1$_a$**. Here, the isomer geometries were optimized in batches, where, starting with the third degree of chlorination, only the n best geometries were used to seed the next generation of structures in the algorithm, n starting at 20 and incrementally increasing to 50 at a substitution level of 11. To obtain reasonable energy diagrams, the ranking of the most favorable isomers per degree of chlorination is limited to direct derivatives of the best isomer of the previous degree of chlorination, both for **Dim-1$_a$** and HBC. Under this constraint, the best isomer structure per degree of chlorination according to GFN2-xTB was then optimized using DFT to yield the energy diagrams shown in **Fig. S19** (**Dim-1$_a$**) and **Fig. 5** (HBC). Note, that the relative total energies $\Delta E_{tot,n_{Cl}}$ with respect to the number of chlorine atoms in the molecule n_{Cl} displayed in these figures were computed as $\Delta E_{tot,n_{Cl}} = E_{tot,n_{Cl}} + n_{Cl}\left(E_{HCl} - E_{Cl_2}\right) - E_{tot,0}$ to account for the substitution of protons with chlorine, where $E_{tot,n_{Cl}}$ is the total energy of the best structure containing n_{Cl} chlorine atoms and E_{HCl} and E_{Cl_2} are the respective total energies of hydrogen chloride and molecular chlorine, obtained from DFT calculations. For a better understanding, the energy steps between the degrees of chlorination are given in **Table S2** (**Dim-1$_a$**) and **Table S3** (HBC)**.** Illustrations of all the molecular structures that are represented in the two energy diagrams are displayed in **Fig. S20** (**Dim-1$_a$**) and **Fig. S21** (HBC).

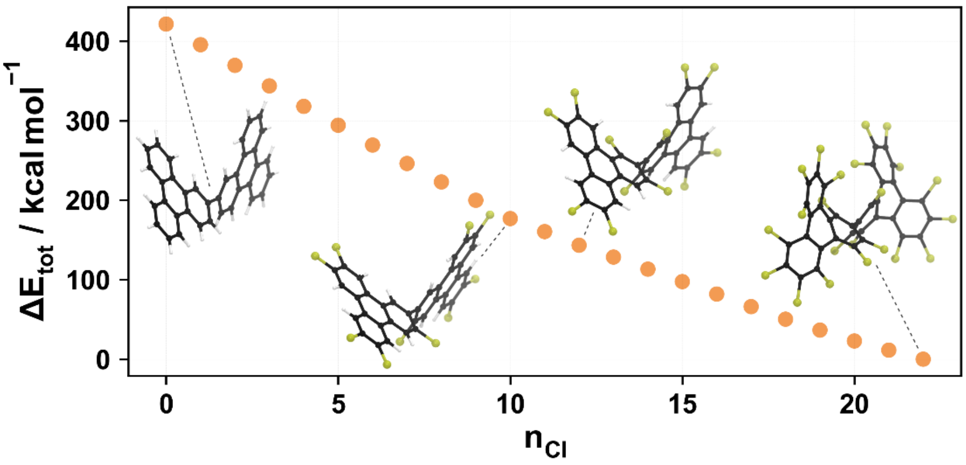

Fig. S19. Relative molecular energies of the most favorable chlorinated isomer derived from Dim-1_a per degree of chlorination, obtained from DFT calculations, as well as the DFT-optimized geometries of the isomers with $n_{Cl} = 0, 10, 12, 22.$

Tab. S2. Molecular energy differences $\Delta E_{tot,i,j} = E_{tot,j} - E_{tot,i}$ between the most favorable chlorinated isomers derived from Dim-1_a.

i → j	*0 → 1*	*1 → 2*	*2 → 3*	*3 → 4*	*4 → 5*	*5 → 6*	*6 → 7*	*7 → 8*	*8 → 9*	*9 → 10*	*10 → 11*
$\Delta E_{tot,i,j}$ / kcal mol^{-1}	-25.99	-25.98	-25.80	-25.76	-23.75	-24.91	-23.20	-23.18	-23.05	-23.04	-16.40
i → j	*11 → 12*	*12 → 13*	*13 → 14*	*14 → 15*	*15 → 16*	*16 → 17*	*17 → 18*	*18 → 19*	*19 → 20*	*20 → 21*	*21 → 22*
$\Delta E_{tot,i,j}$ / kcal mol^{-1}	-17.14	-14.88	-15.20	-15.66	-15.65	-15.82	−15.82	−13.70	−13.68	−11.52	−11.39

Tab. S3. Molecular energy differences $\Delta E_{tot,i,j} = E_{tot,j} - E_{tot,i}$ between the most favorable chlorinated isomers derived from HBC.

i → j	*0 → 1*	*1 → 2*	*2 → 3*	*3 → 4*	*4 → 5*	*5 → 6*	*6 → 7*	*7 → 8*	*8 → 9*
$\Delta E_{tot,i,j}$ / kcal mol^{-1}	-26.07	-25.97	-25.74	-25.66	-25.44	-25.38	-15.15	-16.22	-15.88
i → j	*9 → 10*	*10 → 11*	*11 → 12*	*12 → 13*	*13 → 14*	*14 → 15*	*15 → 16*	*16 → 17*	*17 → 18*
$\Delta E_{tot,i,j}$ / kcal mol^{-1}	-15.52	-15.73	-16.77	-14.90	-14.84	-14.81	-15.16	-15.08	-15.01

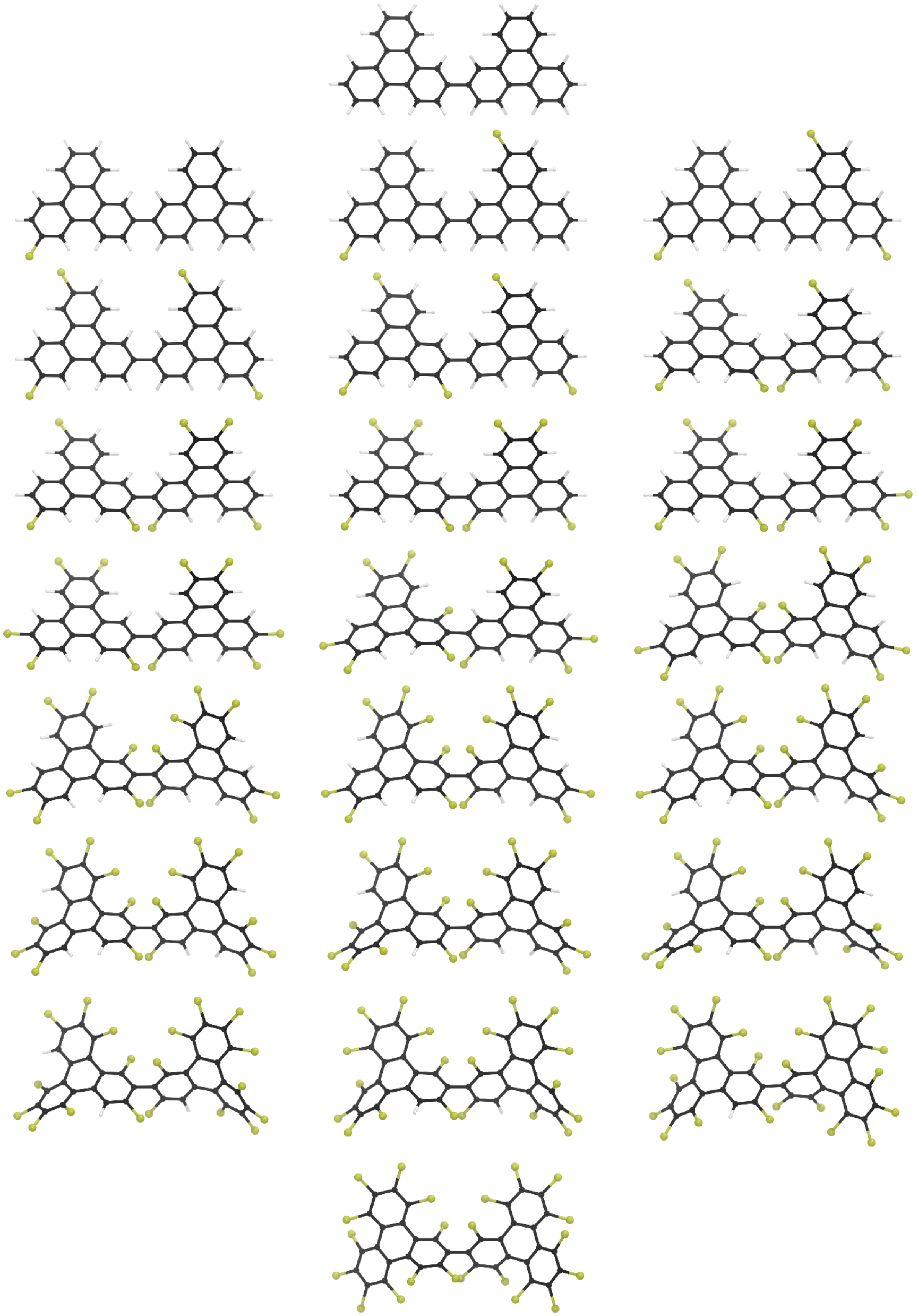

Fig. S20. Molecular structures of the most favorable chlorinated isomer derived from Dim-1_a per degree of chlorination, obtained from DFT calculations.

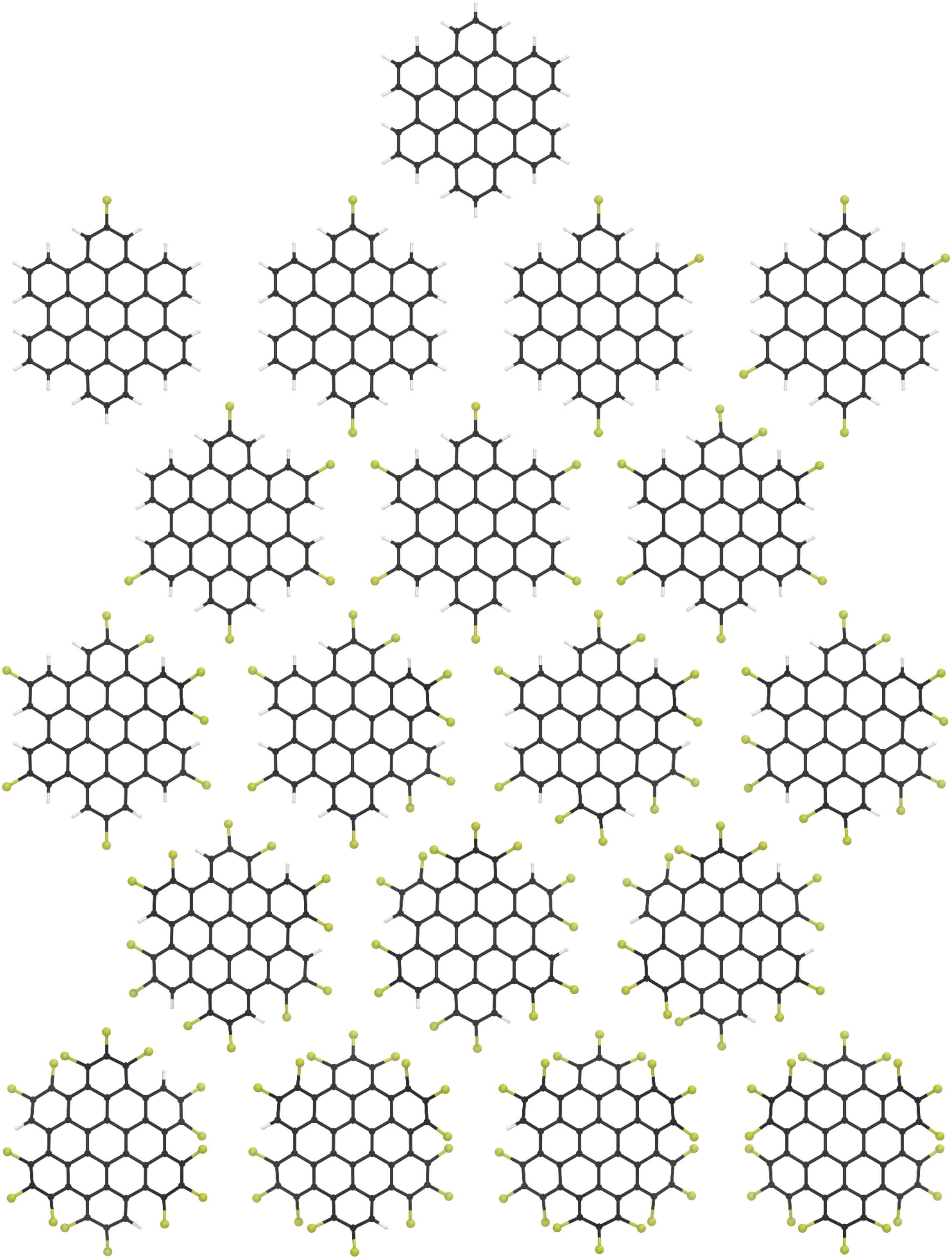

Fig. S.21 Molecular structures of the most favorable chlorinated isomer derived from HBC per degree of chlorination, obtained from DFT calculations.

8 Author contributions

**Daniel M. Baier**	Preparation of the manuscript, mechanochemical reactions and sample purifications, IR, XRD and UV/VIS measurements
**Sven Grätz**	Preparation of the manuscript, mechanochemical reactions and sample purification, UV/VIS measurements
**Babak Farhadi Jahromi**	Preparation of the manuscript, Performing the GFN2-xTB and DFT calculations
**Sarah Hellmann**	Mechanochemical reactions and sample purification
**Konrad Bergheim**	Mechanochemical reactions and sample purification
**Wilm Pickhardt**	MALDI-TOF measurements
**Rochus Schmid**	Preparation of the manuscript
**Lars Borchardt**	Project coordination and supervision

9 References

1 L. Konnert, A. Gauliard, F. Lamaty, J. Martinez and E. Colacino, *ACS Sustain. Chem. Eng.*, 2013, **1**, 1186.
2 S. Grätz, D. Beyer, V. Tkachova, S. Hellmann, R. Berger, X. Feng and L. Borchardt, *Chem. Commun.*, 2018, **54**, 5307.
3 A. Maghsoumi, A. Narita, R. Dong, X. Feng, C. Castiglioni, K. Müllen and M. Tommasini, *Phys. Chem. Chem. Phys.*, 2016, **18**, 11869.
4 N. M. O'Boyle, M. Banck, C. A. James, C. Morley, T. Vandermeersch and G. R. Hutchison, *J. Cheminformatics*, 2011, **3**, 1.
5 C. Bannwarth, S. Ehlert and S. Grimme, *J. Chem. Theory Comput.*, 2019, **15**, 1652.
